# Recent advances in polydopamine-coated metal–organic frameworks for cancer therapy

**DOI:** 10.3389/fbioe.2025.1553653

**Published:** 2025-04-01

**Authors:** Jingchao He, Guangtian Wang, Yongfang Zhou, Bin Li, Pan Shang

**Affiliations:** ^1^ Institute of Translational Medicine, Medical College, Yangzhou University, Yangzhou, China; ^2^ Key Laboratory of the Jiangsu Higher Education Institutions for Nucleic Acid and Cell Fate Regulation, Yangzhou University, Yangzhou, China; ^3^ Teaching Center of Pathogenic Biology, School of Basic Medical Sciences, Harbin Medical University, Harbin, China; ^4^ Department of Oncology, Jining Cancer Hospital, Jining, China; ^5^ Department of Biochemistry and Molecular Biology, Medical College, Guangxi University of Science and Technology, Liuzhou, China; ^6^ Department of Obstetrics and Gynecology, The Affiliated Taizhou People’s Hospital of Nanjing Medical University, Taizhou, China

**Keywords:** metal–organic frameworks, polydopamine, metal–organic frameworks modified with polydopamine, cancer, diagnosis and therapy

## Abstract

The creation and development of classical multifunctional nanomaterials are crucial for the advancement of nanotherapeutic treatments for tumors. Currently, metal–organic frameworks (MOFs) modified with polydopamine (PDA) are at the forefront of nanomedicine research, particularly in tumor diagnostics and therapy, owing to their exceptional biocompatibility, expansive specific surface area, multifaceted functionalities, and superior photothermal properties, which led to significant advancements in anti-tumor research. Consequently, a range of anti-cancer strategies has been devised by leveraging the exceptional capabilities of MOFs, including intelligent drug delivery systems, photodynamic therapy, and photothermal therapy, which are particularly tailored for the tumor microenvironment. In order to gain deeper insight into the role of MOFs@PDA in cancer diagnosis and treatment, it is essential to conduct a comprehensive review of existing research outcomes and promptly analyze the challenges associated with their biological applications. This will provide valuable perspectives on the potential of MOFs@PDA in clinical settings.

## 1 Introduction

Nanomedicine protects drugs, improves drug targeting, enables intelligent drug release, and overcomes drug resistance (Wang and Zhang, 2023). Nanomedicine utilizes the tumor microenvironment to treat tumors and enables comprehensive treatment and diagnosis of tumors, improving the efficiency of tumor treatment (Wang and Zhang, 2023). Nanomedicine plays a pivotal role in tumor diagnosis and therapy, emerging as a frontier for the discovery of innovative diagnostic and therapeutic approaches that are increasingly attracting research interest (Wang and Zhang, 2023; Zhang J. et al., 2023; Forgham et al., 2024). It has enhanced the efficacy of cancer treatment and reduced treatment-associated toxicities, markedly improving the quality of life and survival rates for cancer patients and contributing to the advancement of precision medicine (Sandbhor et al., 2024). Multifunctional nanomaterials are the main elements of nanomedicine and are the carriers of nanomedicine for the diagnosis and treatment of tumors (Zhou L. L. et al., 2024). The design and fabrication of these multifunctional nanomaterials are crucial for achieving efficient and safe tumor treatment and represent a central milestone in the progression of nanomedicine, which can catalyze the development of new diagnostic and therapeutic strategies (Sharma and Otto, 2023; Zhou Z. et al., 2021; Lan et al., 2023). Consequently, the pursuit of multifunctional nanomaterials in the domain of tumor diagnostics and therapeutics, through a comprehensive review, holds the potential to offer insights for the creation of novel multifunctional nanomaterials and foster the discovery of innovative anti-tumor strategies, thus proving to be of significant value.

Metal–organic frameworks (MOFs) with excellent properties are mesoporous crystalline materials composed of metal ions and organic ligands, which have become leading materials in the field of tumor diagnosis and therapy and have received increasing attention (Ma et al., 2023; Binaeian et al., 2023; Wang Y. et al., 2025a). Compared with traditional nanomaterials, MOFs mainly have the following advantages (Wang S. et al., 2024; Shi P. et al., 2023; Gulati et al., 2023; Moharramnejad et al., 2023; Mehata et al., 2023): (1) MOFs possess abundant mesopores and a high specific surface area, enabling efficient drug loading. Some MOFs can degrade within the tumor microenvironment to realize the precise release of drugs at the tumor site. Therefore, MOFs are highly efficient carriers for the construction of nano-drug delivery systems. (2) Some MOFs are composed of metal ions that are essential to the human body and low-toxicity organic ligands, which exhibit good biocompatibility. Some MOFs are degradable, which reduces the long-term toxicity of the nanoparticles. Thus, MOFs have good biosafety, which is the basis for anti-tumor applications. (3) Due to the diverse and multifunctional nature of the metal ions and organic ligands, MOFs exhibit highly efficient imaging properties [computed tomography (CT) imaging, magnetic resonance imaging (MRI), and fluorescence imaging (FI), among others] and therapeutic properties [chemodynamic therapy (CDT), photodynamic therapy (PDT), and sonodynamic therapy (SDT), among others]; their rich and adjustable functions make them an efficient platform for achieving diagnostic and therapeutic integration and comprehensive treatment. (4) MOFs have nano-enzymatic activity, which can scavenge the highly expressed glutathione (GSH) in the tumor microenvironment and catalyze the generation of reactive oxygen species (ROS) and O_2_ from endogenous highly expressed H_2_O_2_ to improve the hypoxic state of the tumor microenvironment. Therefore, MOFs can regulate the characteristics of the tumor microenvironment and enhance the tumor therapeutic effect. Therefore, MOFs have important research significance and can promote the development of novel tumor diagnostic and therapeutic strategies.

Polydopamine (PDA), which is produced from dopamine by self-polymerization reaction under oxygen and an alkaline environment, has excellent properties and is a classical nanomaterial that has been widely studied and highly regarded in the field of tumor diagnosis and therapy (Zhao X. et al., 2024; Mao et al., 2024; Menichetti et al., 2024). Compared with other functional materials, PDA has the following main advantages (Wu H. et al., 2022a; Li M. et al., 2023; Acter et al., 2023; Witkowska et al., 2023; Li H. et al., 2021): (1) PDA not only has good biocompatibility but also has degradability. Therefore, PDA has superior biosafety, which is the basis for its wide application. (2) PDA is an excellent photothermal agent, with high photothermal conversion efficiency and high photothermal stability, demonstrating high efficiency of photothermal performance. (3) PDA has high adhesion and could be coated on the surface of a variety of functional materials, making it an ideal unit for the preparation of multifunctional nanomaterials. (4) The surface of PDA is rich in reactive groups such as catechol, amine, and amino, which not only chelate metal ions (Gd^3+^, Mn^2+^, and Fe^3+^, etc.) to produce T_1_-weighted MRI but also facilitate surface modification. (5) The preparation process of PDA is simple, making it easy to obtain samples for biomedical applications. Therefore, PDA is of great research significance and has broad applications in tumor therapy and diagnosis.

MOFs@PDA could combine the advantages of MOFs and PDA, which is a classic multifunctional material for achieving a good synergistic effect of “1 + 1>2.” MOFs and PDA have both advantages and disadvantages. Some MOFs have problems such as poor water solubility, unstable physiological environments, and lack of active groups on the surface, which limit their biological applications (Zhou et al., 2022). By virtue of its strong adhesion properties, PDA can grow on the surface of MOFs and improve the water solubility, stability, and biocompatibility of MOFs (Zhou et al., 2022). PDAs alone are relatively monofunctional and cannot provide highly efficient tumor diagnosis and treatment. However, PDA can chelate the metal ions that compose MOFs, leading to the growth of MOFs with multiple functions on its surface; this process endows PDA with multifunctionality to meet high clinical therapeutic requirements (Chen L. et al., 2019). Therefore, MOFs@PDA is a classical combination that can overcome the inherent defects of MOFs and PDA and produce new physicochemical properties, which is more suitable for tumor diagnosis and treatment. For example, PDA is coated on the surface of MOFs that are efficiently loaded with drugs, placing the drugs inside the composite material, avoiding the problem of drug leakage in the bloodstream that exists with most carriers, and mitigating the toxic side effects of the drugs (Huang et al., 2023).

**FIGURE 1:**
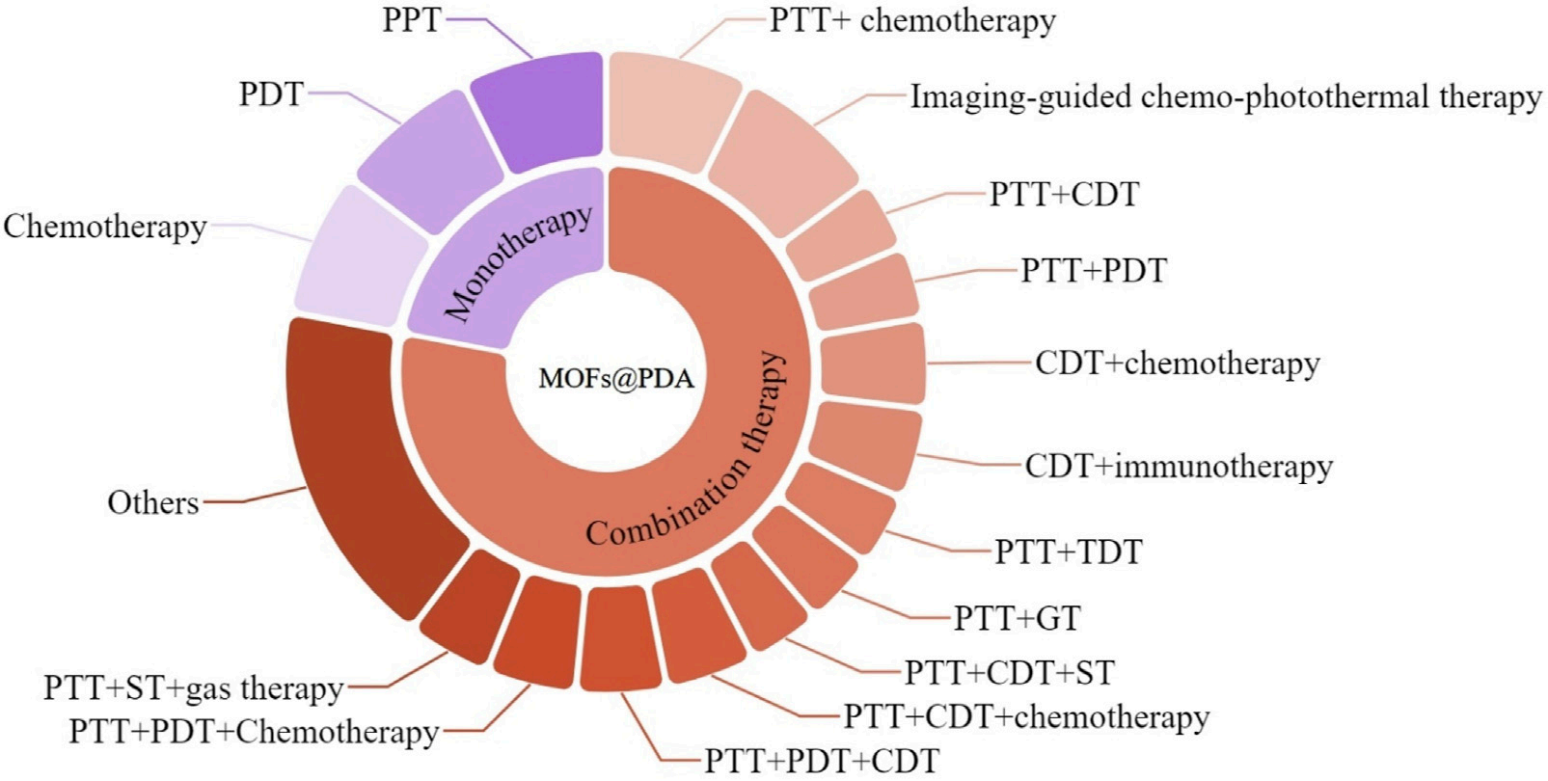
Schematic illustration showing the application of MOFs@PDA in tumor therapy.

Currently, MOFs@PDA has achieved a series of breakthrough research results in the field of tumor diagnosis and treatment by virtue of its excellent performance, which is useful for reference. Unfortunately, no review on MOFs@PDA in the field of tumor diagnosis and therapy has been reported. In this paper, we summarized the application of MOFs@PDA in tumor monotherapy and combination therapy (Figure 1) and analyzed the challenges of MOFs@PDA in oncology applications in a timely manner to deepen the understanding of MOFs@PDA, which is expected to be useful for the construction of new classical multifunctional nanomaterials and the development of novel anti-tumor strategies.

## 2 Application of polydopamine-coated metal–organic frameworks in cancer therapy

MOFs@PDA combine the unique advantages of both MOFs and PDA, overcoming the inherent limitations of each while acquiring new physicochemical properties. This results in a highly efficient and safe classical multifunctional nanomaterial. MOFs@PDA can act as a contrast agent and be used for photodynamic and photothermal therapy, thus achieving the integration of diagnosis and treatment (Guo et al., 2020). MOFs@PDA can efficiently load proteases and drugs, which could achieve comprehensive treatment, and it has made significant breakthroughs in addressing drug resistance, tumor microenvironment-induced therapeutic resistance, and the low efficiency of low-temperature PTT (Li J. M. et al., 2023; Liu G. et al., 2020; Yu H. et al., 2022; Deng X. et al., 2023). The application of MOFs@PDA in cancer diagnosis and therapy is shown in Table 1.

**TABLE 1:** Application of MOFs@PDA in cancer diagnosis and therapy.

MOFs@PDA-based nano-system	Surface modification of MOFs@PDA	Imaging performance	Treatment mode	Reference
DOX@ZIF-HA	HA	T_1_-weighted MRI	Chemotherapy	Shu et al. (2018)
PDA@CPT@MIL-53 (Fe)	_	T_2_-weighted MRI	Chemotherapy	Zhou et al. (2022)
UIO@Ca-Pt	_	_	PDT	Ren et al. (2021)
MnCoO-PDA-PEG-Ce6	PEG	T_2_-weighted MRI and FI	PDT	Wang et al. (2019a)
PDA-Pt@PCN-FA	FA	FI	PDT	Wang D. et al. (2018)
Cu-BTC@PDA	_	T_1_-weighted MRI	PTT	Thirumurugan et al. (2023)
MCP-PEG-RGD	PEG and cRGD	T_1_-weighted MRI	PTT	Wang X. S. et al. (2018)
SOR@ZIF-8@PDA	_	_	PTT + chemotherapy	Hu et al. (2023)
BA@ZIF-8-PDA-PEG	PEG-NH_2_	_	PTT + chemotherapy	Gao S. S. et al. (2023)
DOX/Pd@ZIF-8@PDA	_	PAI	PTT + chemotherapy	Zhu et al. (2019)
PDA-PCM@ZIF-8/DOX	Tetradecanol	_	PTT + chemotherapy	Wu et al. (2018)
PDA/MTX@ZIF-8	_	_	PTT + chemotherapy	Yin et al. (2022)
IDa-PRMSs@ZF	FA	_	PTT + chemotherapy	Zhang W. et al. (2023)
DOX@MoS_2_-PMA	HA	T_1_-weighted MRI and PAI	PTT + chemotherapy	Yang et al. (2021)
DI@HMONs-PMOFs	PEG-NH_2_	T_1_-weighted MRI and PAI	PTT + chemotherapy	Chen et al. (2019)
ZIF-8/DMPP	PEG	T_1_-weighted MRI and PAI	PTT + chemotherapy	Guo et al. (2020)
Fe3O4-NH_2_@PDA@Au@MIL101-NH_2_-DOX	_	T_2_-weighted MRI	PTT + chemotherapy	Li S. et al. (2021)
Gd/Tm- PB@ZIF-8/PDA-DOX	_	T_1_–T_2_ weighted MRI and FI	PTT + chemotherapy	Xu et al. (2020)
BTTP-MOF@PDA/DOX	BTTP	T_1_-weighted MRI	PTT + chemotherapy	Wang Y. T. et al. (2022)
Cu-BTC@PDA	_	_	CDT + PTT	Liu L.et al. (2023)
PDA@Cu/ZIF-8	_	_	CDT + PTT	An et al. (2020)
HG-MIL@PDA	HA	_	CDT + PTT	Wu et al. (2021)
MP@PI	_	FI	CDT + PTT	Deng et al. (2022)
Gd-PDA-Ce6@Gd-MOF	_	T_1_–T_2_ weighted MRI and PAI	PTT + PDT	Pu et al. (2021)
PDA-MB-CAT-ZIF-8	_	_	PTT + PDT	Feng et al. (2020a)
MCDP@Bif	_	PET/CT	CDT + chemotherapy	Li J. M. et al. (2023)
CQ/FA-PDA@MOF	FA	_	CDT + immunotherapy	Chiang B. et al. (2023)
MPDA/AIPH@ZIF-8/GA	_	_	PTT + TDT	Deng et al. (2023)
PDAs-siRNA-ZIF-8	_	PAI and near-infrared imaging	PTT + GT	Feng et al. (2020b)
Zr/Ce-MOFs/GOx/PDA	_	_	PTT + catalytic therapy	You et al. (2024)
TPZ/PFA@UiO-66@PDA	_	PAI	PTT + hypoxia-activated chemotherapy	Chen et al. (2021)
MPDA@ZIF-8/DOX + GOx	_	_	PTT + starvation therapy	Liu et al. (2020)
ZDZP@PP	mPEG-NH_2_	_	PDT + chemotherapy	Ren et al. (2020)
PDA-MOF-E-M	Osteosarcoma cell membranes	T_1_-weighted MRI	PTT + ferroptosis	Liu Y. J. et al. (2024)
PDA@C3N4@MIL/GOx@HA	HA	_	PTT + ST + CDT	Yu et al. (2022)
MGH	HA	PAI	PTT + ST + CDT	Zhang et al. (2019)
Oxa@MIL-PDA-PEGTK	NH_2_-PEGTK-COOH	_	PTT + CDT + chemotherapy	Huang et al. (2022)
RBCM-HCPT@Cu/ZIF-8@PDA	Erythrocyte membrane	_	PTT + CDT + chemotherapy	Ren et al. (2024)
CMC/OSA/PCN-224 (Cu) at PDA	_	_	PTT + PDT + CDT	Zhang and Yuan. (2024)
AIPH/PDA@CuS/ZIF-8	_	PAI	PTT + PDT + CDT	Zhang et al. (2021)
PCN-DOX@PDA	_	T_2_-weighted MRI	PTT + PDT + chemotherapy	Chen B. et al. (2023)
Ini@PM-HP	HA	FI + photothermal imaging	PTT + PDT + chemotherapy	Feng et al. (2022)
PCoA@M	Macrophage membranes	_	PTT + ST + GT	Cheng K. et al. (2022)
DGZPNs	_	_	PTT + ST + chemotherapy	Zhan et al. (2023)
Cu@MIL-101@PMTPC	_	_	CDT + PDT immunotherapy + chemotherapy	An et al. (2024)
d-Arg/GOX/TPZ@MOF (Fe)-PDA/Fe^3+^/FA-BSA	FA-BSA	T_1_-weighted MRI	ST + CDT + RT + GT + chemotherapy	Wang et al. (2023)

### 2.1 Polydopamine-coated metal–organic frameworks in monotherapy for tumor

#### 2.1.1 Chemotherapy

At present, chemotherapy is still one of the main clinical treatments for tumors. However, there are some problems such as the non-specific distribution of chemotherapeutic drugs, premature degradation during circulation, and poor water solubility, which limit the efficient application of chemotherapy in clinics (Wang P. et al., 2024). Therefore, it is of great research value to use a simple method to construct a nano-drug delivery system to overcome the abovementioned shortcomings. Shu et al. (2018) encapsulated DOX inside ZIF-8 by the one-step method, coated ZIF-8 surface with PDA, chelated Fe^3+^ with shell PDA, and connected hyaluronic acid (HA) to the surface of the prepared material by the coordination between the carboxyl group of HA and Fe^3+^, resulting in the preparation of DOX@ZIF-HA Due to the presence of HA, DOX@ZIF-HA could actively target tumor tissues with high CD44 receptor expression and become specifically distributed at the tumor site. DOX@ZIF-HA loaded DOX inside the material to prevent premature degradation and leakage of the drug during circulation. Because PDA chelated Fe^3+^, DOX@ZIF-HA had a relaxation rate (R_1_) of 5.57 mM^−1^ s^−1^, which was higher than that of Gd-DTPA, a clinical MRI contrast agent, and it showed excellent T_1_-weighted MRI performance. DOX@ZIF-HA, with a drug-loading capacity of approximately 8.92%, exhibited good stability and pH-responsive drug release performance, which achieved T_1_-weighted MRI-guided active targeted chemotherapy (Shu et al., 2018). Zhou C. et al. (2022) coated the surface of MIL-53 (Fe) loaded with camptothecin (CPT) by PDA to prepare PDA@CPT@MIL-53 (Fe). The surface modification of PDA not only improved the stability, hydrophilicity, and biocompatibility of MIL-53 (Fe) but also prevented the leakage of CPT in blood circulation. PDA@CPT@MIL-53 (Fe) has a drug-loading capacity of 43.07%, and it showed a pH-responsive drug release, which reduced the side effects of chemotherapy. Due to the presence of MIL-53 (Fe), PDA@CPT@MIL-53 (Fe) possessed a transverse relaxation rate of 50 mM^−1^s^−1^ and showed good T2-weighted MRI performance, which achieved imaging-guided chemotherapy (Zhou C. et al., 2022).

#### 2.1.2 Photodynamic therapy

**FIGURE 2:**
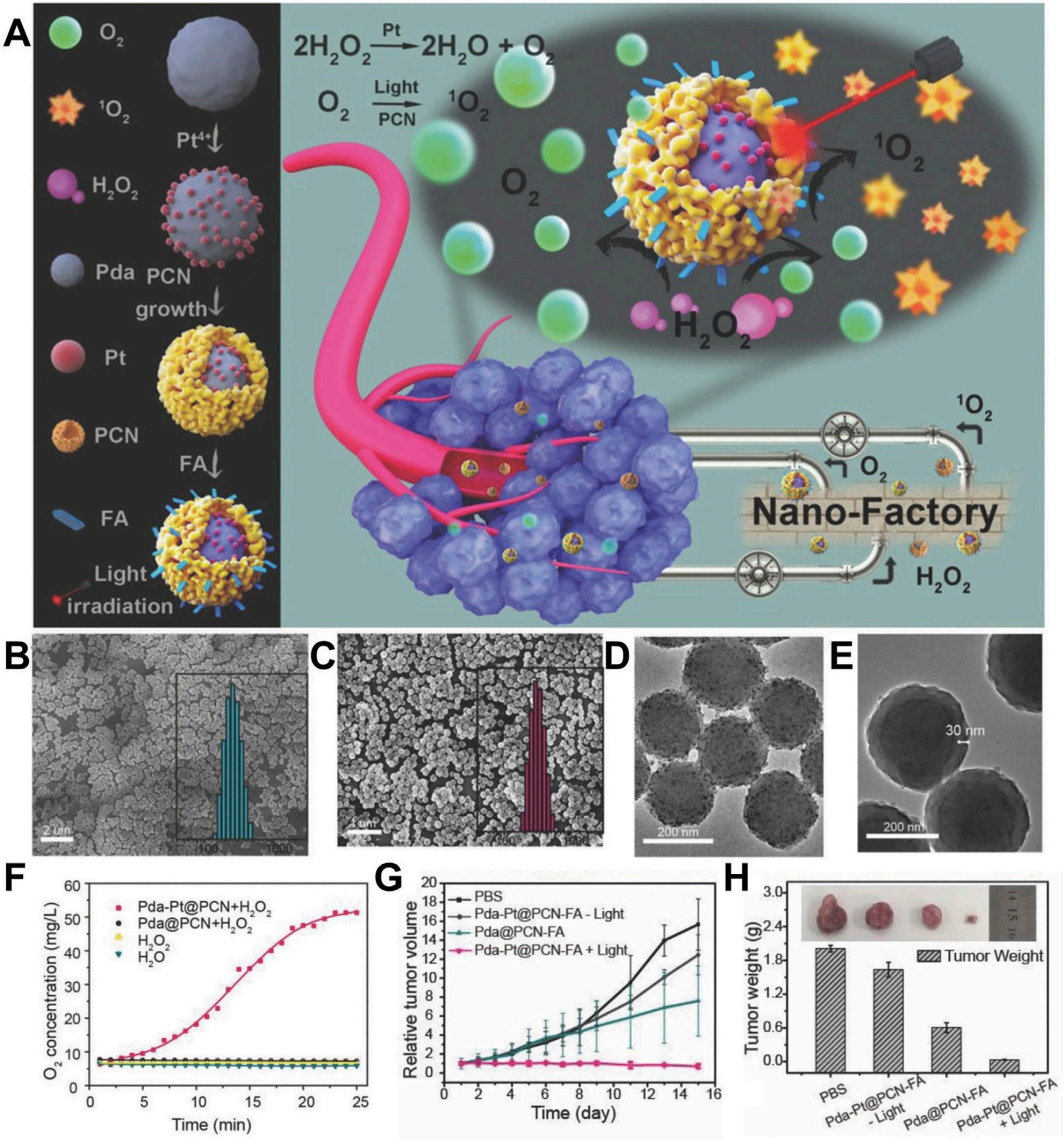
**(A)** Schematic illustration of experimental procedure for preparing PDA-Pt@PCN-FA and anti-tumor mechanisms of PDA-Pt@PCN-FA. DLS and SEM images of **(B)** PDA-Pt and **(C)** PDA-Pt@PCN. TEM images of **(D)** PDA-Pt and **(E)** PDA-Pt@PCN. **(F)** O_2_ generation properties in different groups. **(G)** Relative tumor volume changes in different treatment groups. **(H)** Representative tumor images and average tumor weight of tumor-bearing mice in different treatment groups. Copyright 2018, with permission from Wiley-VCH GmbH and Wang X. S. et al. (2018).

Photodynamic therapy (PDT) can be divided into type I PDT and type II PDT, which has advantages such as non-invasiveness, low side effects, and high tumor specificity, serving as an efficient method for the clinical treatment of tumors (Hsia et al., 2023; Jiang et al., 2023). Most PDT belongs to type II PDT, where the main mechanism consists of photosensitizers converting O_2_ into ROS under light irradiation, which, in turn, destroys tumor cells (Sun et al., 2023; Yu Q.et al., 2024; Yu L. et al., 2024). Tumor hypoxia is the basic feature of a tumor microenvironment and is mainly caused by the rapid increase of tumors and abnormal vascular systems in the tumor, resulting in abnormal blood supply and local hypoxia, which can not only promote the development of tumors but also reduce the efficacy of PDT, chemotherapy, and radiation therapy (Zhang C. et al., 2023; Liu Z. et al., 2024). MOFs@PDA has nano-enzyme activity or can combine with nano-enzyme to catalyze the high concentration of H_2_O_2_ in the tumor to produce O_2_, which significantly improves the photodynamic effect (Ren et al., 2021; Wang D. et al., 2019a; Wang X. S. et al., 2018). Ren et al. (2021) used PDA and CaO_2_ to coat the surface of UIO-66-NH_2_ loaded with photosensitizer TCPP through stirring and applied a Pt nano-enzyme, which grew on the surface of the prepared material by a reduction reaction, resulting in the construction of UIO@Ca-Pt. CaO_2_ reacted with water to form calcium hydroxide and H_2_O_2_, which overcame the deficiency of endogenous H_2_O_2_. Pt could catalyze H_2_O_2_ to produce a large amount of O_2_ and increase the level of O_2_ in the tumor site, thus improving the effect of TCPP-mediated PDT (Ren et al., 2021). The researchers made rational use of nano-enzymes to generate a large amount of O_2_ at the tumor site in a cascade enzyme reaction, alleviating the lack of oxygen at the tumor site and significantly improving the efficiency of PDT, which provided an effective way to overcome the problem of lack of oxygen in the tumor environment. Wang D. et al. (2019a) obtained well-dispersed MnCoO by a one-step method, coated the surface of MnCoO with PDA, modified the shell PDA with PEG, and used the prepared material to load photosensitizer Ce6 through Mn^2+^ and deprotonated COO^−^ coordination, resulting in the preparation of MnCoO-PDA-PEG-Ce6. Due to the presence of PDA and PEG, MnCoO-PDA-PEG-Ce6 showed excellent biocompatibility and good physiological stability, which could be enriched at tumor sites. MnCoO not only had T2-weighted MRI performance but also was a type of nano-enzyme that could continuously catalyze endogenous high concentrations of H_2_O_2_ to produce a large amount of O_2_, thus improving the efficacy of PDT mediated by Ce6. MnCoO-PDA-PEG-Ce6 had a drug-loading capacity of 13.8% and could lead to continuous production of O_2_ *in situ* to improve the state of hypoxia, showing a good effect of PDT on both hypoxic and normoxic tumors (Wang D. et al., 2019a). Wang X. S. et al. (2018) synthesized Pt on the surface of PDA, coated PCN on the surface of PDA, and connected FA with shell PCN, leading to the formation of PDA-Pt@PCN-FA. Because of the stable properties of PDA, researchers could synthesize Pt on the surface of PDA via a reduction reaction. Pt was a type of nano-enzyme that could catalyze the infiltration of H_2_O_2_ into the inner layer of the material and produce a large amount of O_2_, thus improving the efficacy of PDT and inhibiting tumor metastasis. When O_2_ entered the shell PCN, PCN generated a large amount of ROS under laser irradiation, showing highly efficient PDT. PDA-Pt@PCN-FA used nano-enzymes to continuously produce O_2_, which showed high efficiency of PDT for both hypoxic and normoxic tumors (Figure 2) (Wang X. S. et al., 2018). In this study, a core-shell multi-functional complex was reasonably designed, and the different parts of the material had a clear division of labor, performed their own duties, and did not interfere with each other, thus providing a large amount of oxygen for PDT, solving the problem of low efficiency of PDT caused by hypoxia, and inhibiting tumor metastasis (Wang X. S. et al., 2018). The abovementioned studies used nano-enzymes to catalyze the formation of O_2_ from endogenous H_2_O_2_, which improved the efficacy of PDT. However, the insufficient content of endogenous H_2_O_2_ at the tumor site could limit the efficiency of O_2_ production.

#### 2.1.3 Photothermal therapy

Photothermal therapy (PTT) uses photothermal agents to absorb the energy of laser light and convert it into heat, which uses high temperatures to destroy tumor cells (Li N. et al., 2024). PTT has the advantages of high specificity, fewer side effects, high efficiency, and simple operation (Zhang S. et al., 2024; Duan et al., 2023). The multi-functional photothermal agent that integrates diagnosis and photothermal performance can monitor the effect of photothermal therapy and delimit the area of photothermal therapy, which improves photothermal efficiency and is a hot spot in the field of photothermal research (Chen X. et al., 2024; Liu S. et al., 2024; He et al., 2023). Some MOFs have excellent imaging performance, and PDA has excellent photothermal performance. Therefore, MOFs@PDA can serve as a multi-functional photothermal agent, enriching the variety of multi-functional photothermal agents (Thirumurugan et al., 2023; Wang et al., 2018). Thirumurugan et al. (2023) synthesized Cu-BTC by the hydrothermal method and coated the surface of Cu-BTC with PDA to prepare Cu-BTC@PDA. Due to the paramagnetism of Cu^2+^ in Cu-BTC, Cu-BTC@PDA possessed an R_1_ value of 3.01 mg^-1^ s^-1^ and exhibited excellent T_1_-weighted MRI performance *in vitro*. Due to the presence of PDA, Cu-BTC@PDA not only exhibited good biocompatibility but also had 13.32% photothermal conversion efficiency, showing superior photothermal effect (Thirumurugan et al., 2023). Wang et al. (2018) combined Mn_3_ [Co. (CN) _6_]_2_ and PDA into a hybrid nanogel (MCP) by the one-pot method and used MCP to connect PEG and cRGD, thus preparing MCP-PEG-RGD . Due to the presence of PEG and RGD with active targeting performance, MCP-PEG-RGD exhibited good stability, biocompatibility, and long blood circulation time, which could accumulate in large quantities at the tumor site. MCP-PEG-RGD had a photothermal conversion efficiency of 41.3% and possessed an R_1_ value of 5.175 mM^−1^S^−1^, which achieved the active-targeting PTT guided by T_1_-weighted MRI (Wang et al., 2018).

### 2.2 Polydopamine-coated metal–organic frameworks in combination therapy

Because of the heterogeneity, complexity, and diversity of tumors, comprehensive treatment has the advantages of different treatment methods, which is the focus of research in the field of tumor therapy (Turkmen et al., 2024; Yang et al., 2024). MOFs@PDA are multifunctional nanomaterials that combine diagnostic and therapeutic functions, and they are highly efficient platforms for comprehensive treatment, providing a vehicle for exploring novel strategies for tumor diagnosis and treatment and promoting the development of tumor diagnosis and treatment.

#### 2.2.1 MOFs@PDA integrated photothermal therapy and chemotherapy for anti-tumor effects

**FIGURE 3:**
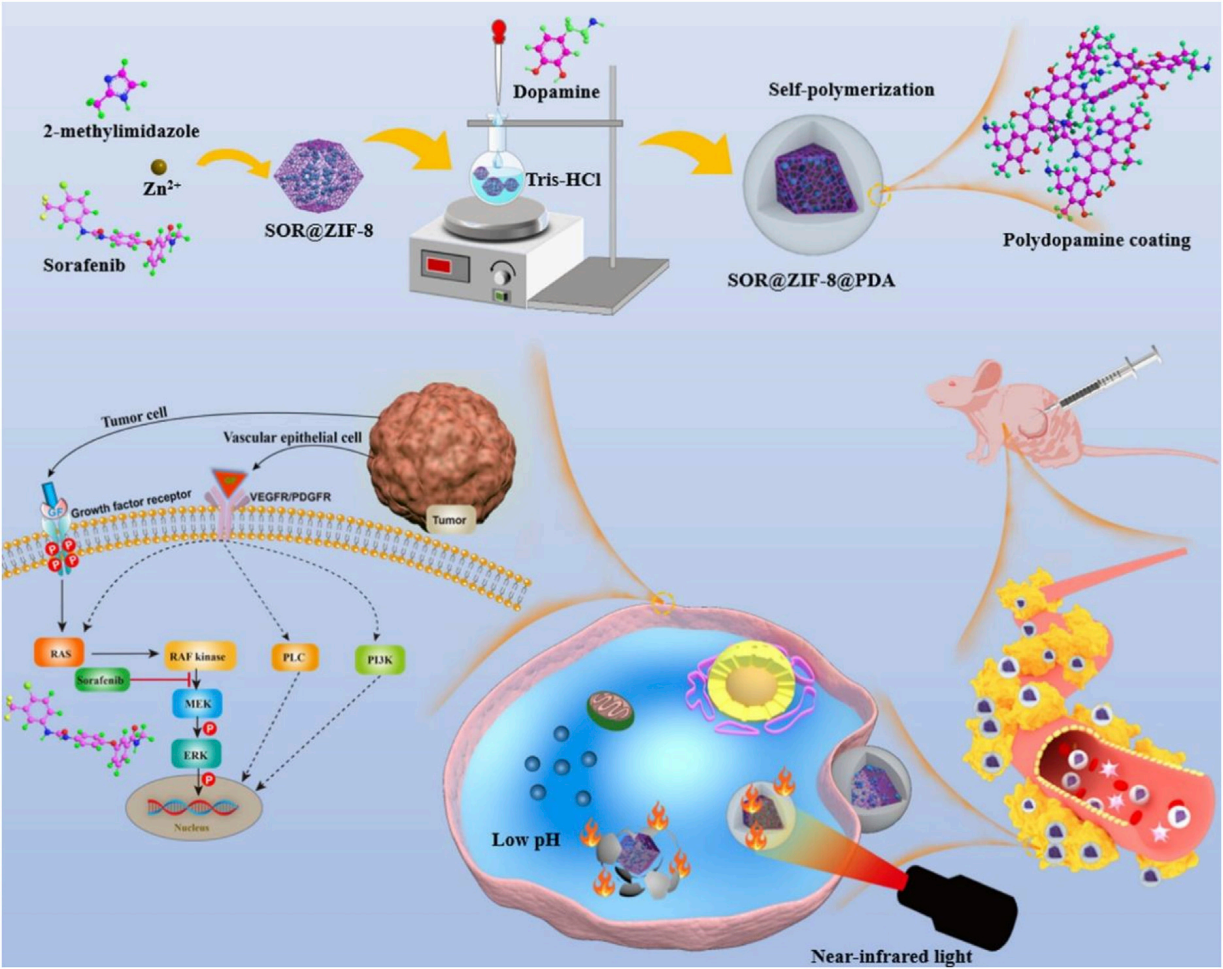
Schematic illustration of the experimental procedure for preparing SOR@ZIF-8@PDA and the combination of photothermal therapy and chemotherapy induced by SOR@ZIF-8@PDA; copyright 2023, with permission from American Chemical Society and Hu et al. (2023).

Chemotherapy can enhance the efficacy of PTT, and PTT promotes drug release and enhances the ability of drugs to enter cells (Yang K. et al., 2023). Therefore, PTT combined with chemotherapy is an efficient combination therapy (Wang X. et al., 2023). At present, liver cancer, with high incidence and mortality, still lacks effective treatment (Ladd et al., 2024). Sorafenib (SOR) is a clinically approved HCC treatment drug, which prolongs the survival time and improves the quality of life of patients (Chon et al., 2024). However, SOR has some problems, such as short half-life, low solubility, easy drug resistance, and adverse side effects, which limit its clinical application (Liu X. et al., 2024). Therefore, many nanomaterials are used to load SOR to improve the therapeutic efficiency of SOR (Wang L. et al., 2023a). However, many nanomaterials load SOR on their surface, which cannot prevent SOR leakage into bloodstream or its toxicity to normal tissues (Kong et al., 2021). In view of this, Hu et al. (2023) encapsulated SOR inside ZIF-8 by a simple one-pot method and coated ZIF-8 with PDA to prepare SOR@ZIF-8@PDA. PDA not only improved the biocompatibility and stability of ZIF-8 but also prevented drug leakage in blood circulation and reduced adverse drug reactions. Due to the presence of PDA, SOR@ZIF-8@PDA had a photothermal conversion efficiency of 23.95%, showing a good photothermal effect. Because ZIF-8 has the characteristics of acid-responsive degradation and PDA accelerated degradation at high temperatures, SOR@ZIF-8@PDA showed pH- and light-stimulated responsive drug release, with the most drug release under both acidic and light conditions. SOR@ZIF-8@PDA exhibited a drug-loading capacity of 7.3% and excellent biosafety, enabling the combination of chemotherapy and PTT, which significantly inhibited tumor growth (Figure 3) (Hu et al., 2023). Due to the self-polymerization of dopamine in an alkaline solution and the coordination between zinc ion (Zn^2+^) and PDA, Gao S. S. et al. (2023) coated PDA on the surface of ZIF-8 loaded with baicalein (BA) and modified PEG-NH_2_ on the surface of shell PDA by electrostatic action to prepare BA@ZIF-8-PDA-PEG. Due to the presence of PDA and PEG-NH_2_, BA@ZIF-8-PDA-PEG possessed good stability, dispersion, and biocompatibility. In an acidic environment, the protonation of the imidazolium group in ZIF-8 resulted in the destruction of coordination between the zinc ion and the imidazole ring, which led to the degradation of ZIF-8. PDA can also undergo degradation through depolymerization under weakly acidic conditions. Therefore, BA@ZIF-8-PDA-PEG showed pH-responsive drug release, facilitating precise drug delivery in the slightly tumor acidic environment of the tumor while reducing the adverse effects of chemotherapy. BA@ZIF-8-PDA-PEG improved the stability and solubility of BA, with a drug-loading capacity of 70.4% and a photothermal conversion efficiency of 39.48%, enabling chemotherapy combined with PTT (Gao S. S. et al., 2023). This study provided a reference for the construction of a nano-drug delivery system based on traditional Chinese medicine and provided a method for the rational use of traditional Chinese medicine. Zhu et al. (2019) encapsulated two-dimensional Pd nanowires and DOX inside ZIF-8 by the one-step method and coated ZIF-8 surface with PDA to prepare DOX/Pd@ZIF-8@PDA. Due to the presence of two photothermal agents, Pd and PDA, DOX/Pd@ZIF-8@PDA had a photothermal conversion efficiency of up to 45% and an excellent photothermal effect. DOX/Pd@ZIF-8@PDA had a drug-loading capacity of 12% and showed pH- and light-stimulated responsive drug release, enabling the combination of PTT and chemotherapy (Zhu et al., 2019). In this study, two-dimensional materials, MOFs, and PDA were combined to obtain novel composites, which improved the drug-loading performance and intelligent drug release ability of two-dimensional materials and made use of the efficient photothermal properties of two-dimensional materials, providing an efficient platform for the realization of comprehensive treatment. Wu Q. et al. (2018) encapsulated DOX inside ZIF-8 by the one-pot method, coated the ZIF-8 surface with phase change materials (tetradecanol and PCM), and applied PDA to coat the surface of PCM@ZIF-8/DOX, leading to the preparation of PDA-PCM@ZIF-8/DOX. Due to the presence of PDA, PDA-PCM@ZIF-8/DOX exhibited good biocompatibility, low toxicity, and high stability, overcoming the problem of rapid degradation of ZIF-8 in an acidic environment. PDA-PCM@ZIF-8/DOX possessed the characteristics of degradation in an acidic environment, showing the pH-responsive release of drugs. PDA-mediated PTT led to local high temperature, resulting in the dissolution of PCM and promoting drug release, which resulted in heat-responsive drug release. Therefore, PDA-PCM@ZIF-8/DOX achieved pH- and thermal-responsive release of drugs, which could enable accurate treatment of tumors and avoid damage to normal tissues. PDA-PCM@ZIF-8/DOX had a drug-loading capacity of 37.86% and a photothermal conversion efficiency of 30.61%, enabling the combination of PTT and chemotherapy (Wu Q. et al., 2018). PDA-PCM@ZIF-8/DOX prepared in this study had pH-responsive degradation properties, which avoided long-term toxicity accumulation of nanomaterials. The thermo-responsive degradation of PCM was used to modify the surface of the material, which enabled the precise release of the drug at the irradiation site of the tumor, promoting the development of precision medicine. Yin et al. (2022) encapsulated methotrexate (MTX) in the inner part of ZIF-8 by the one-pot method and coated ZIF-8 with PDA to prepare PDA/MTX@ZIF-8. PDA/MTX@ZIF-8 had a drug-loading capacity of 16.45% and showed drug release in response to pH and light stimulation, enabling the combination of chemotherapy and PTT (Yin et al., 2022). These studies suggested that ZIF-8@PDA, with its intelligent drug release capability, served as an excellent photothermal agent and an ideal drug carrier, enabling the efficient combination of chemotherapy and PTT. Zhang W. et al. (2023) loaded ammonium bicarbonate (NH_4_HCO_3_) and DOX with rod-shaped mesoporous silica nanoparticles (RMSs), applied PDA to coat RMSs and load photosensitizer ICG, and synthesized ZIF-8 modified with FA on the PDA surface to prepare IDa-PRMSs@ZF. IDa-PRMSs@ZF not only had a photothermal conversion efficiency of 26.06%, showing a good photothermal effect, but also showed drug release in response to pH and light stimulation. FA-ZIF-8 endowed the composites with active tumor targeting and improved their biocompatibility. In an acidic environment and at high temperatures, NH_4_HCO_3_ readily decomposed, producing a large amount of CO_2_, which promoted the rapid release of DOX at the tumor site. This enabled DOX to reach the therapeutic concentration in a short time, thereby improving the effectiveness of chemotherapy. IDa-PRMSs@ZF could actively target the tumor tissue and release several chemotherapeutic drugs within the tumor, thus achieving a highly efficient combination of chemotherapy and PTT. This provided an ideal nano-platform for the construction of a nano-drug delivery system with active targeting capabilities and the ability to trigger drug release in large quantities at the tumor site (Zhang W. et al., 2023).

#### 2.2.2 Imaging-guided chemo-photothermal therapy

**FIGURE 4:**
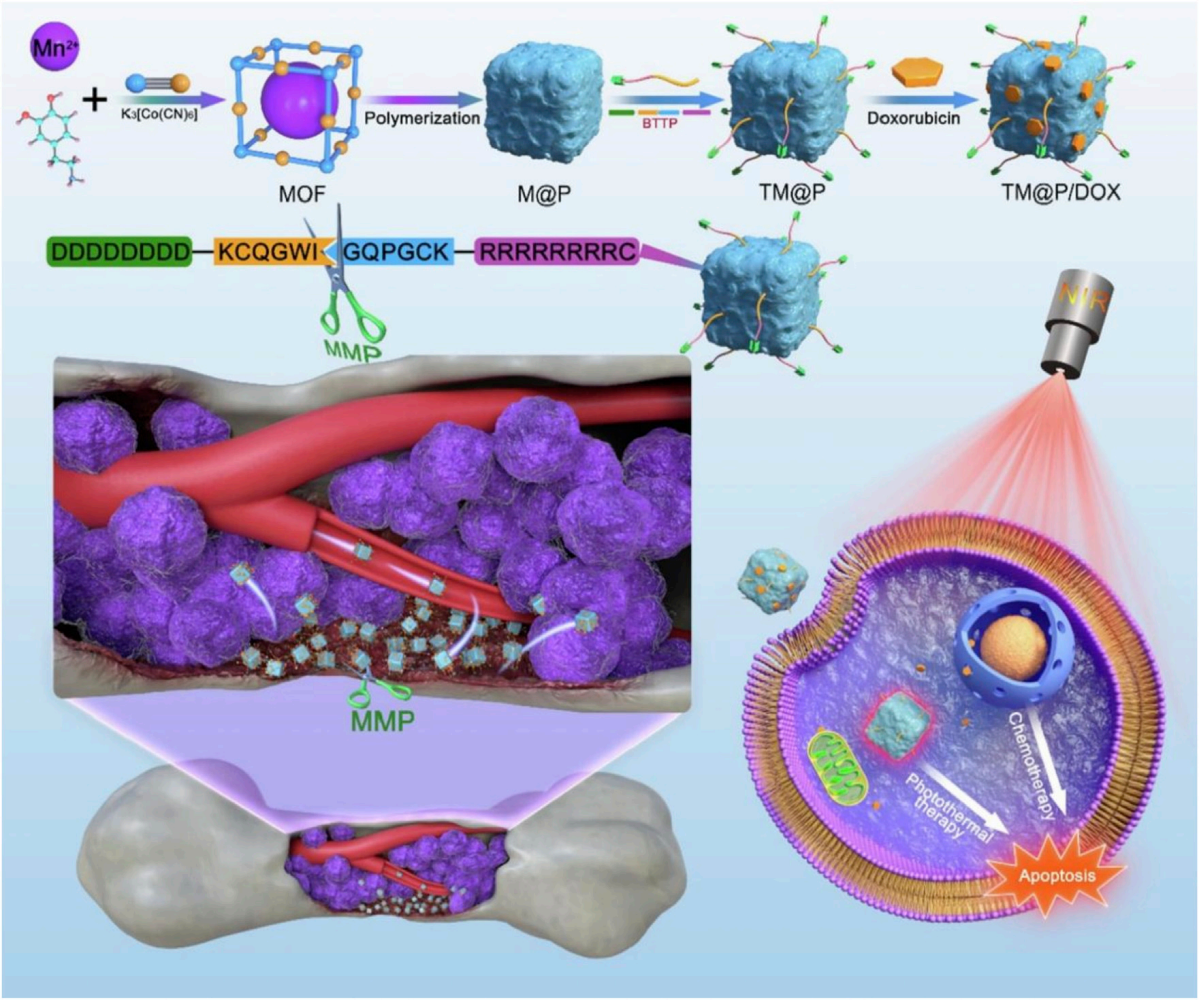
Schematic illustration of experimental procedure for preparing BTTP-MOF@PDA/DOX (TM@P/DOX) and the combination of photothermal therapy and chemotherapy induced by TM@P/DOX with the property of bone tumor cell targeting; copyright 2022, with permission from Elsevier and Wang Y. T. et al. (2022).

Yang S. et al. (2021) synthesized MoS_2_ with photothermal therapy and PA imaging ability by the one-pot method, applied PDA to coat the surface of MoS_2_, used Fe-MOFs consisting of Fe^3+^ and trimesic acid (H_3_BTC) to grow layer-by-layer through self-assembly on the PDA surface, and utilized the Fe-MOFs for DOX-loading and connecting with HA, leading to the preparation of DOX@MoS_2_-PMA. Because the active group of PDA could chelate Fe^3+^, Fe-MOFs could grow on the surface of MoS_2_@PDA, which showed T_1_-weighted MRI properties. The DOX@MoS_2_-PMA could actively target breast cancer cells with high HA receptor expression and had a drug-loading capacity of 21.46%, enabling drug release in response to both pH and light stimulation. DOX@MoS_2_-PMA achieved MRI and PAI-guided the combination of chemotherapy and PTT, which significantly inhibited tumor growth (Yang S. et al., 2021). MoS_2_, as a non-mesoporous material, was combined with mesoporous MOFs to form a composite that leveraged the photothermal properties of MoS_2_ and the drug-carrying capacity of MOFs; this combination compensated for the low drug-carrying efficiency of MoS_2_, enabling highly efficient combination therapy. Chen L. et al. (2019) used PDA to coat hollow mesoporous organosilica nanoparticles (HMONs) loaded with DOX to synthesize Fe-MOFs consisting of Fe^3+^ and H_3_BTC on the surface of PDA through the layer-by-layer self-assembly technique and used Fe-MOFs to connect PEG-NH2 and load ICG, leading to the preparation of DI@HMONs-PMOFs. Because of the coordination between PDA and Fe^3+^ and the PAI performance of ICG, DI@HMONs-PMOFs had T_1_-weighted MRI and PAI performance, which was helpful in achieving accurate diagnosis and imaging-guided treatment. DI@HMONs-PMOFs had a drug-loading capacity of 11.88% for DOX and a drug-loading capacity of 19.52% for ICG, and it showed drug release in response to pH and light stimulation, achieving an efficient combination of PTT and chemotherapy (Chen L. et al., 2019). In this study, taking advantage of the fact that PDA was easy to coat on the surface of functional materials and chelate metal ions, HMONs and MOFs were combined to achieve efficient loading of drugs and photothermal agents, achieving comprehensive treatment guided by imaging, which provided a novel idea for the construction of a multi-functional complex. Guo et al. (2020) encapsulated DOX inside ZIF-8 by the one-pot method, coated the ZIF-8 surface with PDA, chelated Mn^2+^, and connected PEG with shell PDA, leading to the preparation of ZIF-8/DMPP. Due to the chelation of Mn^2+^ by PDA, ZIF-8/DMPP exhibited T_1_-weighted MRI and PAI performance, improving imaging accuracy. ZIF-8/DMPP with a drug-loading capacity of 18.9% exhibited pH-responsive degradation and showed pH- and light stimulation-responsive drug release, achieving a combination of chemotherapy and PTT (Guo et al., 2020). This study illustrated that MOFs@PDA, with PDA as the shell, could directly chelate metal ions to achieve imaging capabilities through a simple method; this provided a simple and rational strategy for preparing multifunctional nano-platforms with integrated diagnostic and therapeutic functions. Li S. et al. (2021) used Au nanocages, a photothermal agent, to adhere to the core-shell structure of Fe_3_O_4_-NH_2_@PDA through physical stirring; they then synthesized MIL-101-NH_2_ on the surface of the synthetic material by the microwave thermal method and loaded DOX into the MIL-101-NH_2_ shell, leading to the preparation of Fe_3_O_4_-NH_2_@PDA@Au@MIL101-NH_2_-DOX. Fe_3_O_4_-NH_2_@PDA@Au@MIL101-NH_2_-DOX exhibited good biocompatibility, exhibited drug release in response to light stimulation, and enabled T_2_-weighted MRI-guided chemotherapy combined with PTT (Li S. et al., 2021). These studies suggest the correctness of the following. Due to its strong adhesive properties, PDA can be easily coated on the surface of different types of materials (Wu H. et al., 2022a; Li M. et al., 2023; Acter et al., 2023; Witkowska et al., 2023; Li H. et al., 2021). Since PDA could chelate the metal ions that constitute MOFs, MOFs easily grow on the PDA surface. Through the bridge action of PDA, MOFs were easy to combine with different types of nanomaterials to form a complex, which provided multi-functional platforms for the integration of diagnosis and treatment. Xu et al. (2020) synthesized ZIF-8 on the Gd^3+^- and Tm^3+^-doped Prussian blue (Gd/Tm-PB) surface, coated the ZIF-8 surface with PDA, and loaded DOX with shell PDA, leading to the preparation of Gd/Tm-PB@ZIF-8/PDA-DOX. Due to the presence of Gd^3+^ and Tm^3+^, Gd/Tm-PB showed the performance of T_1_–T_2_-weighted MRI and fluorescence imaging (FI), leveraging the complementary advantages of different imaging modes to improve diagnostic accuracy. Due to the degradation of PDA at high GSH concentration and the degradation of ZIF-8 in acidic environments, the complex achieved drug release in response to GSH and pH. Gd/Tm-PB@ZIF-8/PDA-DOX enabled the combination of chemotherapy and PTT guided by multimodal imaging (Xu et al., 2020). Given the tumor microenvironment’s characteristics—low pH and high GSH concentration—the complex demonstrated potential for accurate drug release at the tumor site, thus reducing the toxicity of chemotherapy (Xu et al., 2020). The bone microenvironment also provided a “barrier” for malignant bone tumors, preventing chemotherapeutic drugs and tumor-targeting molecules from entering bone tumor cells (Natoni et al., 2019; Liu et al., 2014; Chen et al., 2017; Tian et al., 2021). Therefore, it is of great research value to develop new efficient and safe methods for the treatment of bone tumors. Wang et al. (2022a) prepared the complex of Mn–Co MOFs and PDA by the one-step method, used PDA to connect bone-targeted small molecule BTTP through the Michael addition reaction, and applied PDA to efficiently load DOX through π–π stacking, leading to the preparation of BTTP-MOF@PDA/DOX. Due to the presence of BTTP, BTTP-MOF@PDA/DOX could actively target bone tumors and efficiently enrich nanomaterials and DOX at bone tumor sites. BTTP-MOF@PDA/DOX had a drug-loading capacity of 9.23%, showed pH-responsive drug release, and possessed a photothermal conversion efficiency of 42.67%, showing a good photothermal effect. Due to the Mn^2+^ in the Mn–Co MOFs, the BTTP-MOF@PDA/DOX had excellent T_1_-weighted MRI performance, enabling the integration of diagnosis and treatment. BTTP-MOF@PDA/DOX could actively target bone tumors, and it achieved an efficient combination of PTT and chemotherapy to inhibit bone tumor growth and bone destruction, which provided ideas for overcoming low treatment efficacy caused by the bone microenvironment (Figure 4) (Wang et al., 2022a).

#### 2.2.3 MOFs@PDA integrate photothermal therapy and chemodynamic therapy to enhance anti-tumor effects

**FIGURE 5:**
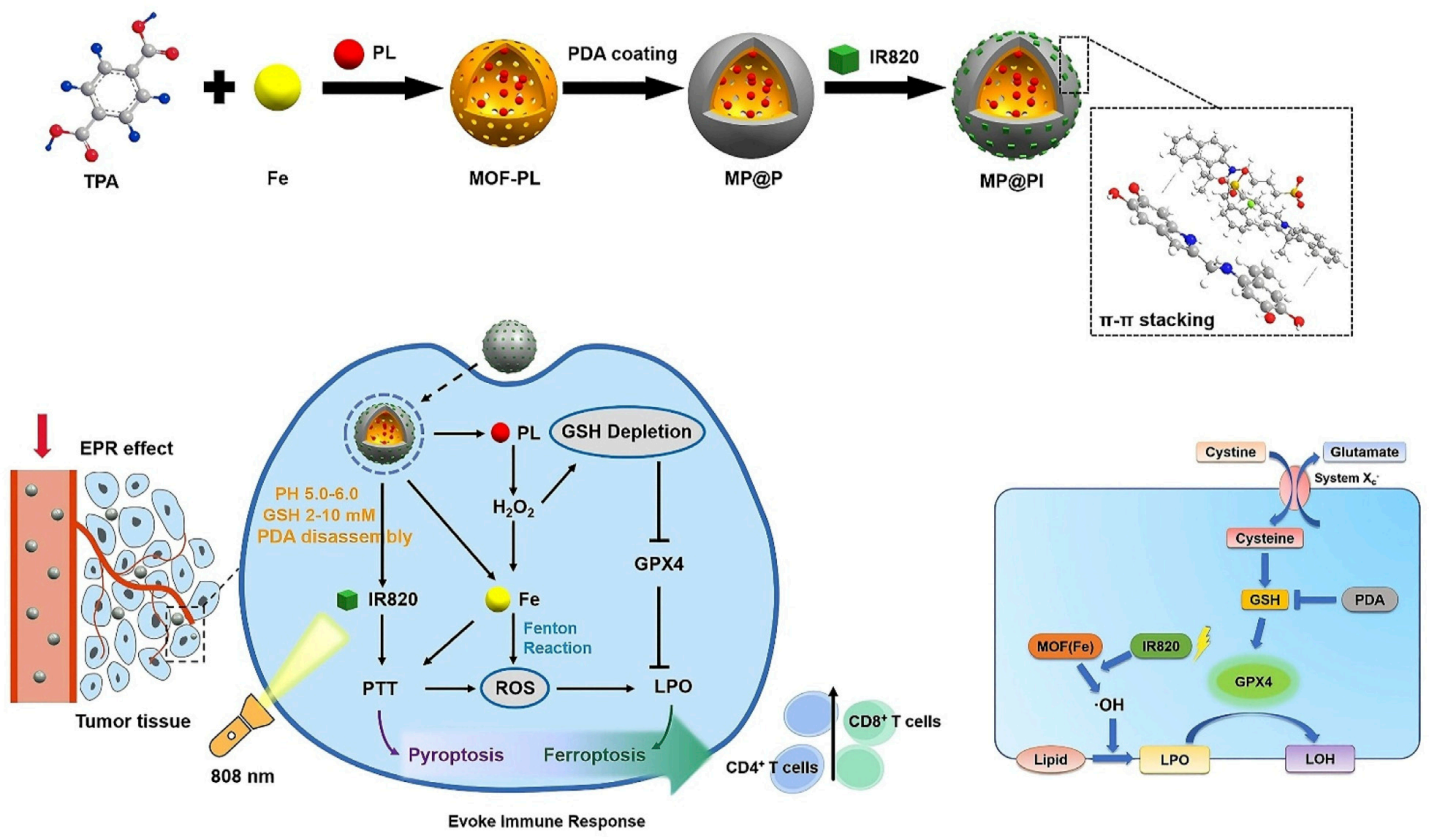
Schematic illustration of the fabrication of MP@PI and the combination of photothermal therapy and chemodynamic therapy induced by MP@PI with the mechanism of ferroptosis; copyright 2022, with permission from the American Chemical Society and Deng H. et al. (2022).

Chemodynamic therapy (CDT) involves metal ions reacting with H_2_O_2_ in the tumor through the Fenton reaction to generate toxic hydroxyl radicals (•OH), which destroy tumor cells (Cheng B. et al., 2023). Insufficient intracellular H_2_O_2_ concentration leads to less ROS production, and high GSH expression in tumor cells scavenged ROS, which reduced the efficiency of CDT (Cheng B. et al., 2023). The combination of PTT and CDT is an excellent therapeutic modality (Cheng B. et al., 2023; Yan et al., 2024; Gao Q. et al., 2023). PTT generated high temperatures that increased the efficacy of the Fenton reaction and promoted the effect of CDT, and CDT generated ROS that inhibited the activity of heat shock proteins and enhanced the sensitivity of PTT (Yan et al., 2024; Gao Q. et al., 2023). Liu L. et al. (2023) coated PDA on the surface of Cu-BTC composed of Cu^2+^ and H_3_BTC to prepare Cu-BTC@PDA. The shell PDA improved the stability and biocompatibility of Cu-BTC. The photothermal conversion efficiency of Cu-BTC@PDA was 49.38%, which was significantly higher than that of PDA (36.12%). The photothermal performance of Cu-BTC@PDA was enhanced by the plasma generated by the d–d leap of Cu^2+^ in Cu-BTC. Cu-BTC@PDA released Cu^2+^ in the acidic microenvironment. Cu^2+^ consumed GSH and generated Cu^+^, which reduced the clearance of OH. Cu^+^ reacted with H_2_O_2_ in tumor cells to generate OH, which produced CDT. Cu-BTC@PDA achieved highly efficient PTT combined with CDT, which significantly inhibited the growth of melanoma (Liu L. et al., 2023). An P. et al. (2020) applied Cu^2+^-doped ZIF-8 to grow on the surface of PDA, leading to the preparation of PDA@Cu/ZIF-8. Cu^2+^ released by PDA@Cu/ZIF-8 could scavenge GSH and produce Cu^+^, which could react with H_2_O_2_ through the Fenton reaction to form OH, resulting in CDT. PDA-mediated PTT promoted GSH depletion and enhanced Fenton reaction efficiency, thereby disrupting intracellular redox balance and improving the efficacy of CDT. PDA@Cu/ZIF-8 could effectively deplete GSH, facilitating strong energy between PTT and CDT (An P. et al., 2020). In view of the low efficiency of CDT caused by the insufficient concentration of endogenous H_2_O_2_, Wu et al. (2021) synthesized MIL-101-NH_2_ composed of Fe^3+^ and 2-aminoterephthalic acid on the surface of PDA modified by PVP and applied shell MIL-101-NH_2_ to load GOx and connect HA, leading to the preparation of HG-MIL@PDA. Due to the presence of HA, HG-MIL@PDA could actively target tumor cells with high expression of CD44 receptors and prolong blood circulation time, thus efficiently enriching the tumor site. Due to the degradability of shell MIL-101-NH_2_, HG-MIL@PDA has the dual stimulation of pH and light to release Fe^3+^ and GOx. GOx could catalyze glucose to produce H_2_O_2_ and gluconic acid in the presence of O_2_, which could compensate for the deficiency of endogenous H_2_O_2_. Fe^3+^ could react with GSH to deplete it and generate Fe^2+^, which inhibited the antioxidant system and reduced ROS clearance. Fe^2+^ reacted with a large amount of H_2_O_2_ to produce a large amount of ROS, thus achieving high-efficiency CDT. Gluconic acid could reduce the acidic environment of the tumor site, which promoted the efficiency of the Fenton reaction and improved the effect of CDT. HG-MIL@PDA had a photothermal conversion efficiency of 26.03%, showed a good photothermal effect, and enabled an efficient combination of CDT and PTT (Wu et al., 2021). In this study, HG-MIL@PDA had the ability to generate a large amount of H_2_O_2_ and remove GSH, solving the problem of CDT inefficiency due to the insufficient endogenous H_2_O_2_ and high expression of GSH, which could provide a new idea for the efficient use of CDT in the treatment of tumors (Wu et al., 2021). Different from apoptosis, necrosis, and pyroptosis, ferroptosis has a mode of death characterized by iron-dependent lipid peroxidation and the accumulation of large amounts of reactive oxygen species, which can promote the efficacy of chemotherapy, radiotherapy, PDT, and PTT (Ye et al., 2024). Studies have shown that the inhibition of cystine/glutamate transporter (System xc-) and glutathione peroxidase 4 (GPX4) is the main mechanism leading to ferroptosis (Song et al., 2024). The inhibition of GSH, that is, the GPX4 cofactor, leads to the suppression of the GPX4 expression, which leads to lipid peroxidation and the generation of ferroptosis (Song et al., 2024; Yang et al., 2025; Nie et al., 2024). Deng H. et al. (2022) coated PDA on the surface of MOFs loaded with piperlongumine (PL) and incorporated IR 820 using shell PDA, leading to the formation of MP@PI. PDA could consume GSH and reduce the expression of GPX4, which was beneficial for the occurrence of ferroptosis and enhanced the efficacy of CDT. PI produced a large amount of H_2_O_2_ in the tumor site, which could overcome the problem of poor efficacy of CDT caused by the deficiency of endogenous H_2_O_2_. A large number of iron ions in MOFs could react with H_2_O_2_ to produce a large amount of ROS, which led to lipid peroxidation, promoted the generation of ferroptosis, and produced efficient CDT. PTT mediated by PDA and IR820 not only promoted the efficiency of CDT but also produced the effect of pyroptosis. Under laser irradiation, MP@PI could clear GSH and downregulate the expression of GPX4, resulting in lipid peroxidation, the generation of ferroptosis, and CDT. MP@PI showed pH-responsive drug release and FI capability, and it effectively combined ferroptosis and pyroptosis to eliminate tumors, achieving an efficient combination of PTT and CDT (Figure 5) (Deng H. et al., 2022).

#### 2.2.4 PTT + PDT

**FIGURE 6:**
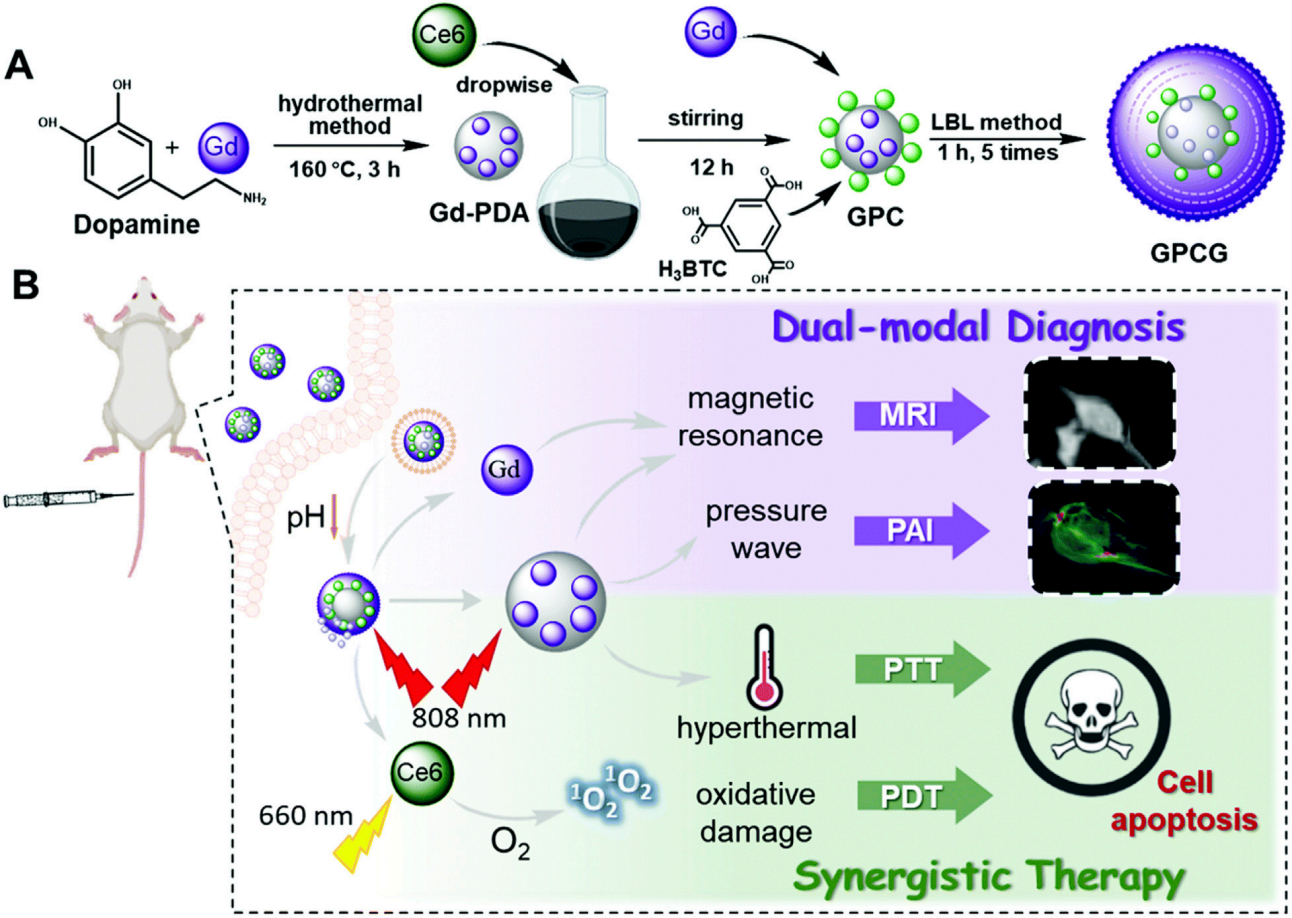
**(A)** Schematic representation of synthesis of Gd-PDA-Ce6@Gd-MOF. **(B)** the application of Gd-PDA-Ce6@Gd-MOF for efficient combination of photodynamic therapy and photothermal therapy guided by MR/PA imaging. copyright 2021, with permission from the Royal Society of Chemistry and Pu et al. (2021).

Both PTT and PDT are effective local therapies, which can produce a good synergistic anti-tumor effect (Zhang M. et al., 2024a). Pu et al., (2021) synthesized gadolinium (III) ion-doped PDA using a simple hydrothermal method, loaded photosensitizer Ce6 onto Gd-PDA by electrostatic interaction and π–π interaction, and coated Gd-MOF on the surface of Gd-PDA NPs using layer-by-layer self-assembly technology, leading to the preparation of Gd-PDA-Ce6@Gd-MOF. Due to the presence of shell Gd-MOF, Gd-PDA-Ce6@Gd-MOF exhibited good stability, enabled pH- and light-stimulated drug release, and prevented premature drug leakage in blood circulation. Gd-PDA-Ce6@Gd-MOF had a photothermal conversion efficiency of 39.14% and a drug-loading capacity of 7.4%, and it possessed longitudinal proton relaxation time of 13.72 mM^−1^ S^−1^ and transverse proton relaxation time of 216.14 mM^−1^ S^−1^, enabling PDT and PTT guided by MRI and PAI (Pu et al., 2021). Feng J. et al. (2020a) coated ZIF-8 on the surface of PDA and encapsulated photosensitizer MB and catalase (CAT) inside the shell ZIF-8 by the one-step method, leading to the preparation of PDA-MB-CAT-ZIF-8. PDA-MB-CAT-ZIF-8 prevented the leakage of MB and CAT in blood circulation and achieved the responsive drug release of pH, which facilitated precise drug release at the tumor site. CAT catalyzed endogenous H_2_O_2_ to produce a large amount of O_2_, which improved the effect of MB-mediated PDT. PDA-MB-CAT-ZIF-8 has a drug-loading capacity of 3.4% for CAT and 5% for MB, and it solved the problem of lack of oxygen at the tumor site by the rational use of biological enzymes, achieving PTT–PDT combination therapy (Figure 6) (Feng J. et al., 2020a).

#### 2.2.5 CDT+ chemotherapy

**FIGURE 7:**
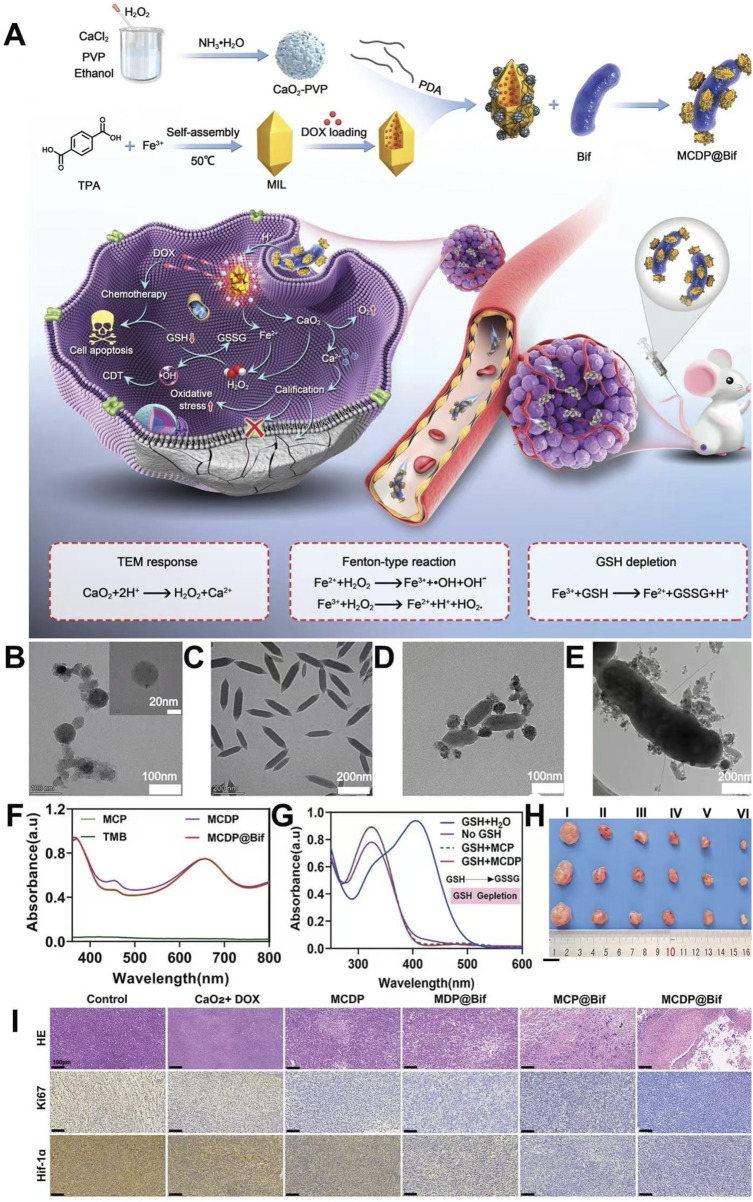
**(A)** Schematic representation of synthesis of MCDP@Bif and synergistic therapy induced by MCDP@Bif. **(B)** TEM image of CaO_2_. **(C)** TEM image of MIL. **(D)** TEM image of MCDP. **(E)** TEM image of the MCDP@Bif biohybrid. **(F)** OH generation in different groups. **(G)** GSH depletion performance in different groups. **(H)** Representative tumor images in different groups. **(I)** HE staining, Ki67 staining, and HIF-1α staining of tumor tissues in different groups. Copyright 2023, with permission from Wiley-VCH GmbH and Li J. M. et al. (2023).

Due to the lack of targeting and abnormal blood supply in the tumor site, traditional chemotherapeutic drugs or nanomaterials could not penetrate the hypoxic area of the solid tumor and accumulate in the hypoxic area at high concentrations, which strongly limited the therapeutic effect of the hypoxic area (Li J. M. et al., 2023). Therefore, the therapeutic effect of tumors could be improved by efficiently and accurately delivering therapeutic drugs or nanomaterials to the tumor area of hypoxia (Li J. M. et al., 2023). Li et al. loaded CaO_2_ and DOX with MIL, coated the surface of the preparation material with PDA, and used shell PDA to adhere to anaerobic *Bifidobacterium infantis* (Bif), leading to the preparation of MCDP@Bif. The PDA coating on the shell could not only prevent the leakage of encapsulated CaO_2_ and DOX but also promote the adhesion of the composite material to Bif. Due to the presence of Bif, MCDP@Bif could actively target the hypoxic area of the tumor and enrich the hypoxic area of the tumor with high concentrations, thus reducing the systemic adverse reactions of DOX. Shell PDA was degraded in a tumor microenvironment of high GSH, low pH, and high ROS, leading to the release of CaO_2_@MIL-DOX from MCDP@Bif. In the tumor acidic microenvironment, CaO_2_@MIL-DOX released CaO_2_, Fe^3+^, and DOX. Fe^3+^ depleted GSH and generated Fe^2+^, which reduced ROS consumption and improved the efficacy of CDT. CaO_2_ produced a large amount of H_2_O_2_ and Ca^2+^ in the tumor acidic microenvironment. Fe^2+^ reacted with H_2_O_2_ to produce a large amount of ROS, resulting in efficient CDT. A large amount of Ca^2+^ released by CaO_2_ caused calcium overload in tumor cells, increased the level of oxidative stress in tumor cells, and promoted apoptosis, thus enhancing the chemotherapeutic effect of DOX. MCDP@Bif utilized anaerobic bacteria to target hypoxic tumor areas, exhibited pH-responsive drug release, and generated large amounts of H_2_O_2_ at the tumor site; this enabled highly effective, low-toxicity CDT-combined chemotherapy, providing novel therapeutic strategies for overcoming treatment resistance caused by hypoxia (Figure 7) (Li J. M. et al., 2023).

#### 2.2.6 CDT + immunotherapy

**FIGURE 8:**
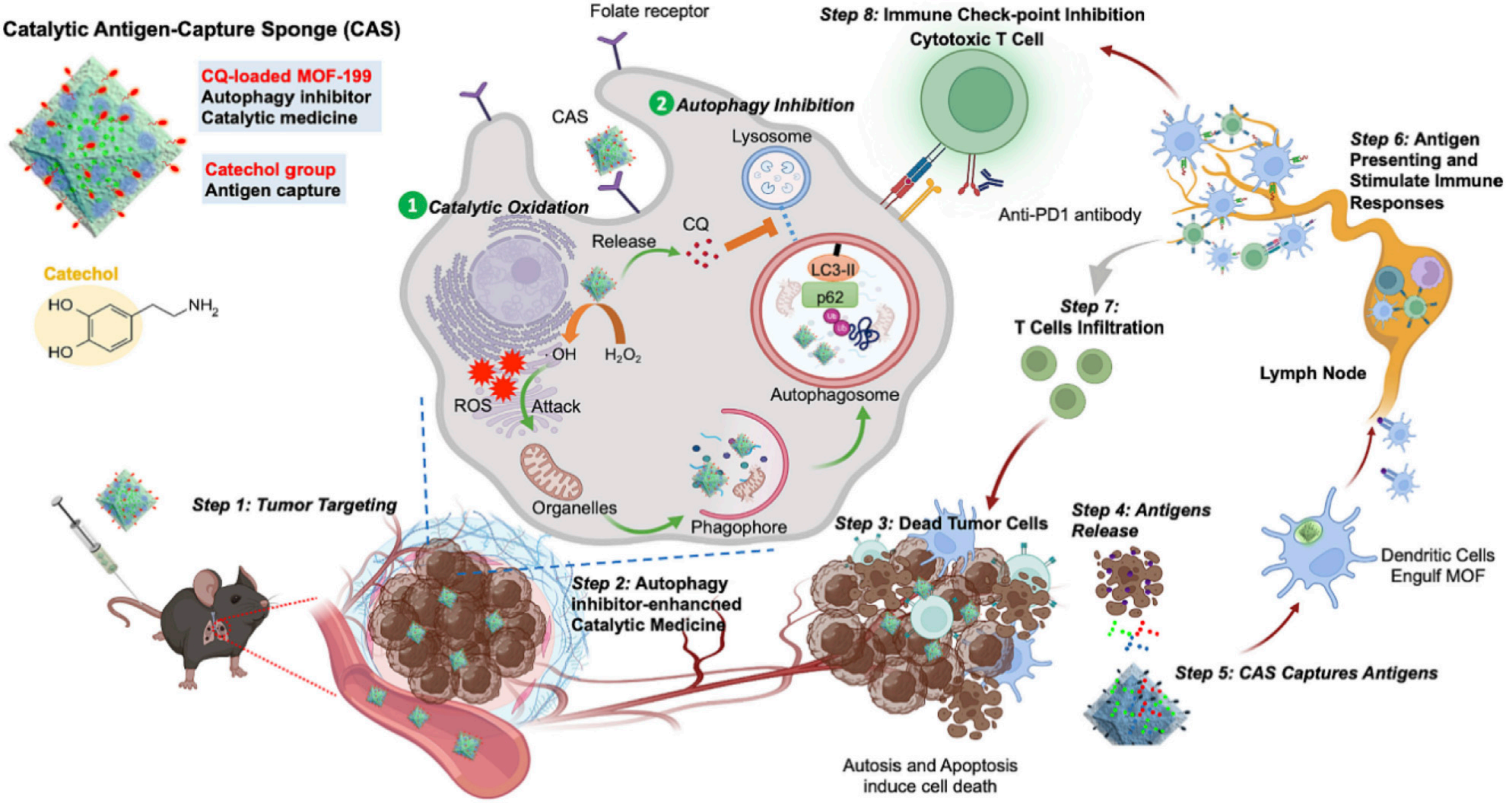
Schematic representation of the high efficacy of immunotherapy induced by catalytic antigen capture sponges (CASs) serving as immunostimulants; copyright 2023, with permission from Elsevier and Chiang et al. (2023). CAS was efficiently enriched at the tumor site by the folate receptor, which led to chemodynamic therapy and inhibited autophagy to generate immunogenic cell death, improving T-cell infiltration. Tumor antigens were presented by dendritic cells, leading to activation of T cells. The immune checkpoint inhibitors were efficiently combined with CAS, improving the efficacy of inhibiting tumor growth.

In immunotherapy, immunogenic cell death (ICD) can activate T cells, thereby destroying tumor cells. ICD results in the release of several damage-related molecular patterns (DAMPs), including high mobility group protein 1 (HMGB1), adenosine triphosphate (ATP), and calmodulin (CRT) from cancer cells (Alavijeh and Akhbari, 2024). Dendritic cells (DCs) can phagocytize and present DAMP and further activate T lymphocytes for immunotherapy (Alavijeh and Akhbari, 2024). The low immunogenicity of tumors and the low efficiency of antigen delivery strongly limited the effect of immunotherapy (Li Q. et al., 2022; Cheng W. et al., 2022). In view of this, Chiang et al. (2023) synthesized MOF-199 using a hydrothermal method, used MOF-199 to load chloroquine (CQ) via π–π interaction, and used PDA to coat on the outside of MOF-199 and attach it with folic acid (FA), leading to the preparation of CQ/FA-PDA@MOF. Cu^2+^ in the complex reacted with GSH to form Cu^+^, which reduced ROS clearance caused by GSH. Cu^+^ reacted with H_2_O_2_ via the Fenton reaction to generate large amounts of ROS, causing CDT and immunogenic death of several tumor cells, which led to the release of DAMP. CQ inhibited protective autophagy generated by CDT, leading to the collapse of cellular self-defense mechanisms, exacerbating cytotoxicity, and promoting the release of tumor-associated antigens. CQ/FA-PDA@MOF could absorb a large number of tumor-associated antigens released by the abovementioned process and transfer them to dendritic cells to induce cytotoxic T-lymphocyte infiltration, enabling efficient delivery of antigens, which resulted in efficient immunotherapy and inhibition of tumor metastasis. CQ/FA-PDA@MOF efficiently produced tumor-associated antigens by CDT, enabled efficient antigen delivery, and achieved a highly efficient combination of CDT and immunotherapy, providing a novel approach to overcome the low immunogenicity and heterogeneity of tumors that resulted in low immunotherapeutic efficacy (Figure 8) (Chiang et al., 2023).

#### 2.2.7 PTT + TDT

**FIGURE 9:**
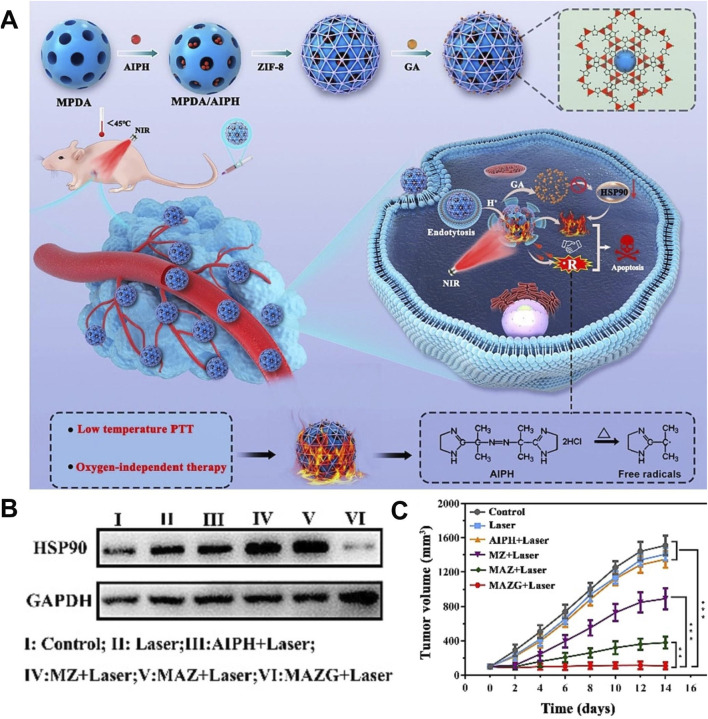
**(A)** Schematic illustration of the synthesis of MPDA/AIPH@ZIF-8/GA and the combination of thermodynamic therapy and photothermal therapy induced by MPDA/AIPH@ZIF-8/GA. **(B)** Inhibition of HSP90 protein expression properties in different treatment groups. **(C)** Tumor volume changes in different treatment groups. Copyright 2023, with permission from Elsevier and Deng X. et al. (2023).

Traditional high-temperature PTT (>50°C) can cause skin damage and induce inflammation, which limits the clinical application of PTT (Li K.et al., 2023). Low-temperature PTT (≤45°C) can avoid potential damage to normal tissues, which has great potential clinical value (Liu W. et al., 2023). However, a large number of heat shock proteins are produced during PTT, which can repair cell thermal damage and significantly reduce the efficiency of low-temperature PTT (Fan et al., 2023). Inhibiting the expression of heat shock protein to achieve low-temperature PTT meets the clinical requirements for efficient and low toxicity treatment, which has important research value (Li X. et al., 2021). Deng X. et al. (2023) prepared MPDA/AIPH@ZIF-8/GA by coating ZIF-8 on the surface of mesoporous dopamine (MPDA) loaded with 2,2-azobis [2-(2-imidazolin-2-yl) propane]-dihydrochloride (AIPH) and using ZIF-8 to load gambogic acid (GA). MPDA/AIPH@ZIF-8/GA improved the stability of AIPH and prevented the leakage of AIPH in blood circulation. Due to the degradation of ZIF-8 in the acidic environment and the degradation of ZIF-8 promoted by high temperature, MPDA/AIPH@ZIF-8/GA achieved drug release in response to pH and light, which reduced the side effects of chemotherapy. Due to the presence of PDA, the complex had a photothermal conversion efficiency of 24.7%, showing a good photothermal effect. The released GA inhibited the expression of HSP90 and reversed the thermotolerance of tumor cells to achieve low-temperature PTT. Under laser irradiation, the large amount of heat generated by the complex led to the rapid decomposition of AIPH to produce oxygen-independent cytotoxic alkyl radicals, which caused oxidative damage to cancer cells, resulting in tumor cell destruction and enabling thermodynamic therapy (TDT). The MPDA/AIPH@ZIF-8/GA exhibited good therapeutic safety and realized low-temperature PTT combined with TDT, which had a significant inhibitory effect on both hypoxic and normoxic tumors (Figure 9) (Deng X. et al., 2023). This study utilized traditional Chinese medicine to efficiently inhibit the expression of heat shock proteins, enabling low-temperature PTT. This approach provides a potential strategy for achieving low-temperature PTT and holds significant reference value. In addition, this study provided an O_2_-independent free radical generation scheme, which enriched the methods of tumor treatment based on free radicals and provided an effective solution for the treatment of hypoxic tumors.

#### 2.2.8 PTT+ GT

**FIGURE 10:**
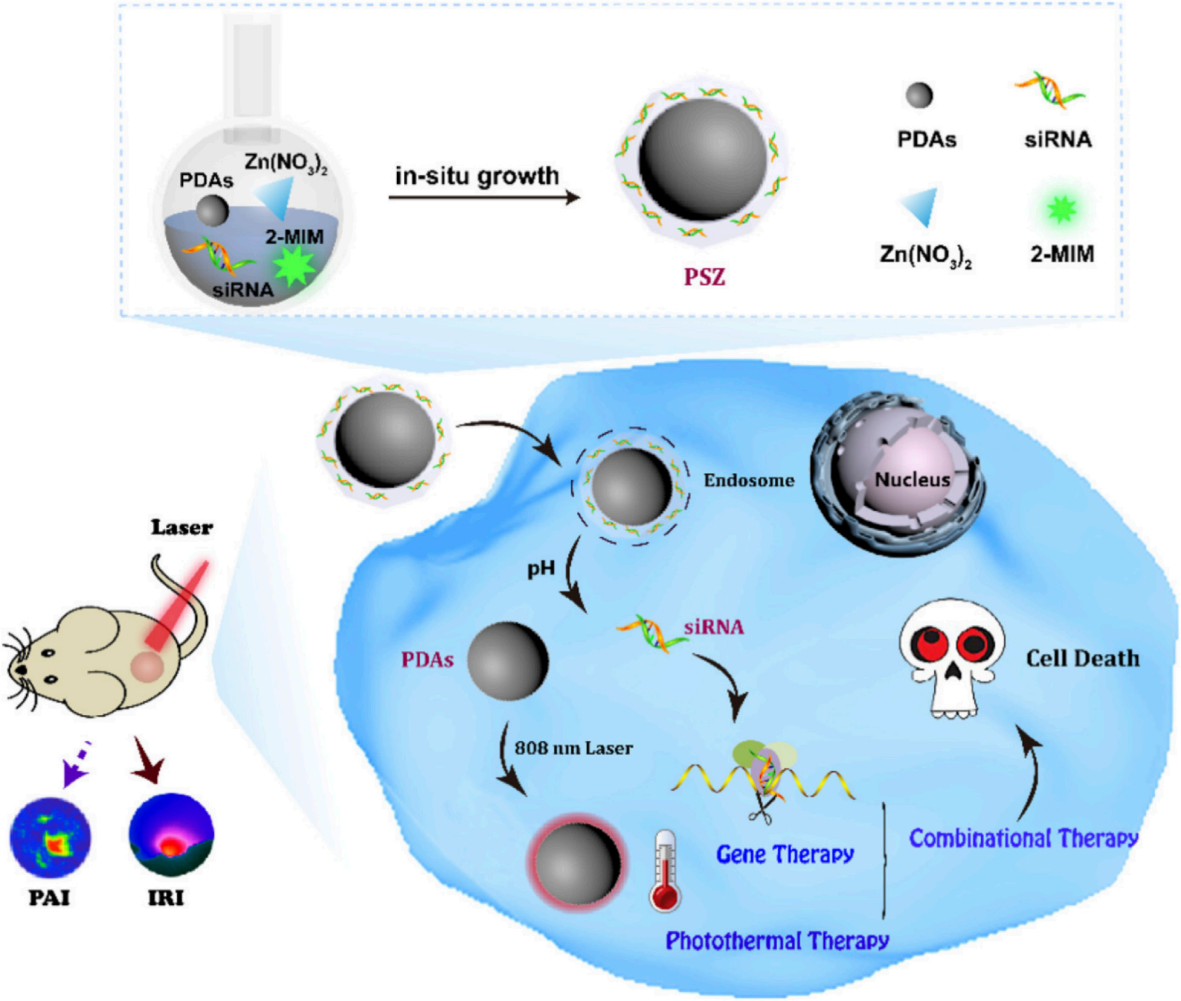
Schematic illustration of the synthesis of PDAs-siRNA-ZIF-8 and the combination of gene therapy and photothermal therapy generated by PDAs-siRNA-ZIF-8; copyright 2020, with permission from the American Chemical Society and Feng J. et al. (2020b).

Small interfering RNA (siRNA)-induced cancer gene therapy (GT), which inhibits the expression of specific genes, has promising applications (Jadhav et al., 2024). However, there are some problems with siRNA, such as nuclease degradation, low cell uptake efficiency, and non-specific biological distribution and immune response, which hinder the clinical application of siRNA (Tao et al., 2024). Therefore, the development of novel nano-carriers for the efficient delivery of siRNA to tumor sites has important research value (Kandasamy and Maity, 2024; Moazzam et al., 2024). Feng J. et al. (2020b) prepared PDA-siRNA-ZIF-8 by coating ZIF-8 on the surface of PDA and encapsulating siRNA inside the shell ZIF-8 by the one-step method. Because the siRNA was inside ZIF-8, PDA-siRNA-ZIF-8 avoided the enzymatic degradation of siRNA and prevented the leakage of siRNA in blood circulation. Due to the acid-responsive degradation of ZIF-8, the complex was not only enriched in the tumor site by the enhanced permeability and retention (EPR) effect but also had the ability to accurately release siRNA in the tumor acidic microenvironment, reducing the adverse side effects of gene therapy. PDA-siRNA-ZIF-8 had a photothermal conversion efficiency of 39%, showed a good photothermal effect, and enabled the combination of PTT and gene therapy guided by PAI (Figure 10) (Feng J. et al., 2020b). In this study, siRNA was encapsulated in nanomaterials, which solved the problems of siRNA and enabled the efficient delivery of siRNA and the accurate release at the tumor site, providing a potential strategy for the construction of a novel siRNA delivery system.

#### 2.2.9 Combination of the other two treatment methods

**FIGURE 11:**
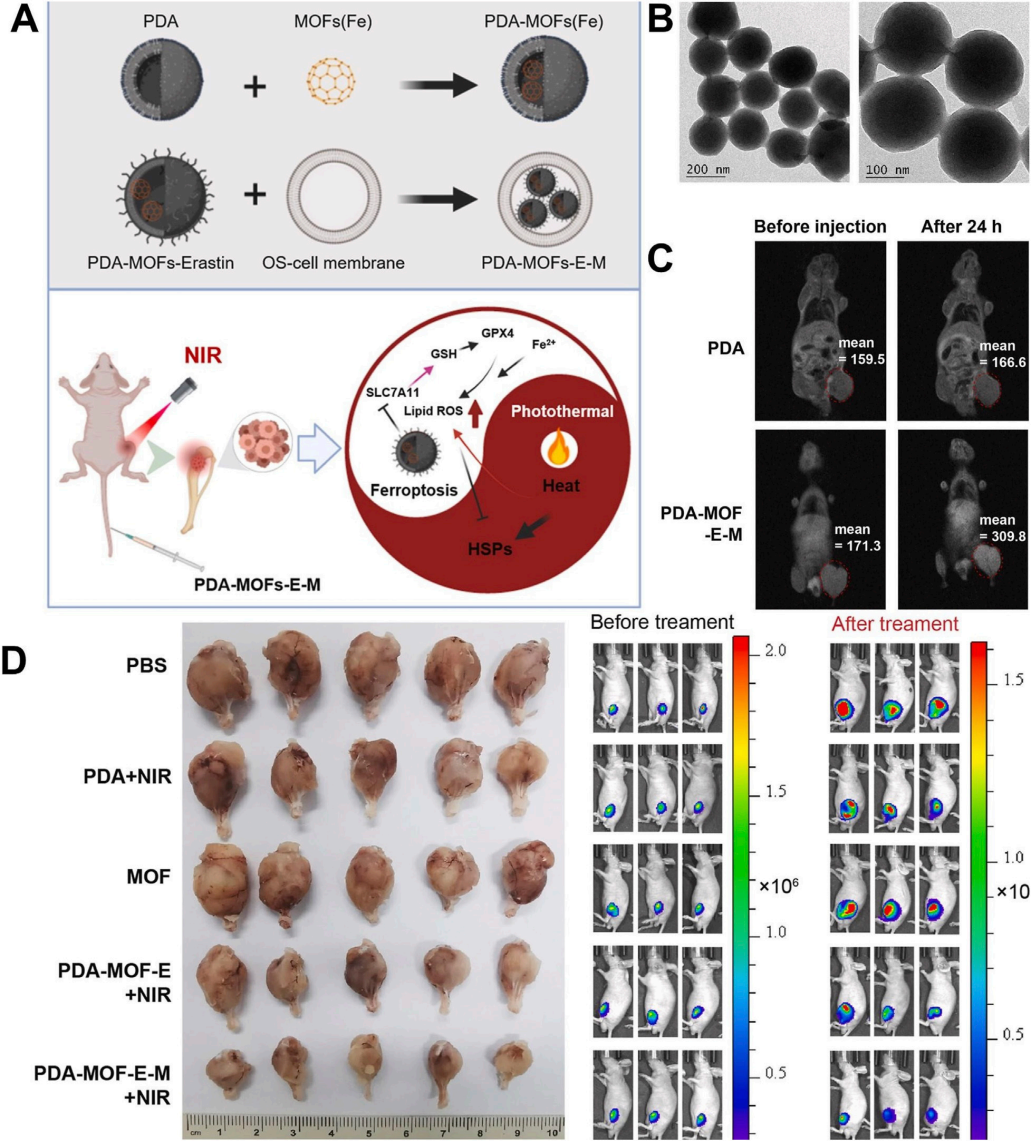
**(A)** Schematic illustration of experimental procedure for preparing PDA-MOF-E-M and the combination of ferroptosis and photothermal therapy generated by PDA-MOF-E-M. **(B)** SEM images of PDA-MOF-E-M. **(C)** T_1_-weighted MRI images of PDA/Fe and PDA-MOF-erastin-M *in vivo*. **(D)** Luminescence images of mice in different treatment groups and tibial tumor images in different treatment groups. Copyright 2024, with permission from KeAi Communications and Liu Y. J. et al. (2024).

Catalytic therapy mainly regulates the tumor microenvironment (TME) through specific catalytic reactions, leading to the depletion of GSH and the production of ROS and oxygen, leading to tumor cell death, which has the advantages of high specificity and low toxicity (Zheng et al., 2025; Bonet-Aleta et al., 2024). However, the high expression of GSH in tumor cells and the insufficient supply of endogenous H_2_O_2_ limit the efficiency of catalytic therapy (Xiong et al., 2024). In view of this, You et al. (2024) loaded glucose oxidase (GOx) with Zr/Ce-MOFs and coated Zr/Ce-MOFs with PDA to prepare Zr/Ce-MOFs/GOx/PDA. GOx catalyzed glucose to produce a large amount of H_2_O_2_, which compensated for the insufficient supply of endogenous H_2_O_2_. Ce^3+^ in the Zr/Ce-MOFs catalyzed H_2_O_2_ to produce highly toxic OH and Ce^4+^. Ce^4+^ could consume GSH and generate Ce^3+^. A large amount of GSH consumption could avoid the consumption of OH produced by catalytic reaction. Due to the presence of PDA, Zr/Ce-MOFs/GOx/PDA had a photothermal conversion efficiency of 26.2% and produced a good photothermal effect, which promoted the efficiency of the catalytic reaction mentioned above. Zr/Ce-MOFs/GOx/PDA achieved a cascade catalytic reaction within the tumor microenvironment to regulate H_2_O_2_ and GSH, enabling PTT combined with catalytic therapy with high efficacy and low toxicity, which provided ideas for designing nano-enzymes to regulate the TME to improve therapeutic efficacy (You et al., 2024). In order to solve the problem of hypoxia in the tumor microenvironment, improving hypoxia is a challenging task (Shen et al., 2023). Researchers can also take advantage of the characteristics of the tumor hypoxic microenvironment to use hypoxia-activated toxic drugs to destroy hypoxic tumors (Zhao L. et al., 2024). Chen H. et al. (2021) used the large surface area and high porosity of UiO-66 to efficiently load perfluorotributylamine (PFA) and tirapazamine(TPZ) and coated PDA on the surface of UiO-66, leading to the preparation of TPZ/PFA@UiO-66@PDA (Chen H. et al., 2021). TPZ/PFA@UiO-66@PDA remained stable in PBS and the culture medium for 24 h and could be enriched in the tumor site by the permeability and retention effect. Due to the presence of PDA, TPZ/PFA@UiO-66@PDA not only exhibited a good photothermal effect but could also prevent early leakage of TPZ and PFA in blood circulation. PFA adsorbed O_2_ in the tumor site, resulting in significant hypoxia of tumor cells and the upregulation of the oxygen-dependent HIF-1α pathway, which led to tumor cell apoptosis. The significant hypoxic environment induced by PFA activated the conversion of TPZ to highly toxic benzotriazine (BTZ), resulting in efficient chemotherapy. TPZ/PFA@UiO-66@PDA exhibited good biocompatibility and enabled the combination of PTT and hypoxia-activated chemotherapy, which effectively inhibited the growth of hypoxic tumors (Chen H. et al., 2021). This study made use of the hypoxia characteristics of the tumor microenvironment and used PFA to adsorb O_2_ from the tumor site to create a significant hypoxia environment, leading to the efficient activation of hypoxia-sensitive drugs and effectively eliminated hypoxia tumors, which provided a strategy for solving the problem of treatment resistance caused by hypoxia (Chen H. et al., 2021). Liu G. et al. (2020) prepared MPDA@ZIF-8/DOX + GOx by growing ZIF-8 *in situ* on the surface of MPDA loaded with DOX and encapsulating GOx inside ZIF-8. The shell ZIF-8 encapsulated GOx inside, which prevented the leakage of GOx in blood circulation, improved the stability of GOx, and ensured the efficient catalytic activity of GOx. In an acidic environment, the outer shell ZIF-8 degraded and released GOx, which consumed glucose and inhibited ATP production, inhibiting the function of ATP-dependent P-glycoprotein (P-gp) transporter proteins. MPDA@ZIF-8/DOX + GOx released GOx and DOX in a sequential manner and released GOx first to inhibit Pgp function and limit the drug efflux, which led to a large accumulation of DOX at the tumor site, thus reversing the resistance. MPDA@ZIF-8/DOX + GOx with a drug-loading capacity of 68.3% showed pH-responsive release of GOx and DOX, which significantly inhibited the growth of breast cancer resistant to DOX and reversed drug resistance (Liu G. et al., 2020). This study constructed a novel nano-drug delivery system that could efficiently load P-gp inhibitors and chemotherapeutic drugs, release P-gp inhibitors and chemotherapeutic drugs sequentially, and effectively reverse drug resistance by inhibiting ATP production, providing a new strategy for overcoming chemotherapeutic drug resistance. Ren et al. (2020) synthesized ZIF-8 on the surface of ZIF-67 loaded with DOX, encapsulated PpIX inside ZIF-8, and used PDA to wrap ZIF-8 and connect mPEG-NH_2_, leading to the preparation of ZDZP@PP. Due to the presence of PDA and mPEG-NH_2_, ZDZP@PP remained stable in PBS and DMEM culture medium for a week and showed excellent biocompatibility, which facilitated the accumulation of nanomaterials at the tumor site. Different parts of ZDZP@PP were loaded with different drugs, which enabled the sequential release of DOX and PpIX and avoided the reaction between different drugs. Because the complex had the characteristics of acid-responsive degradation, ZDZP@PP showed pH-responsive drug release, which was conducive to accurate drug release in the tumor acidic microenvironment. ZIF-67 had the function of nano-enzyme, which could catalyze endogenous H_2_O_2_ to produce O_2_, improving PpIX-mediated PDT. ZDZP@PP utilized nano-enzymes to overcome the problem of oxygen deprivation at the tumor site and achieved a highly effective combination of PDT and chemotherapy (Ren et al., 2020). The combination of PTT and ferroptosis showed excellent synergistic effects, and PTT could enhance the sensitivity of cells to ferroptosis (Zeng et al., 2022; Wu et al., 2022b). Iron ions released by Fe-MOF could catalyze H_2_O_2_ to produce OH through the Fenton reaction, resulting in cell oxidative damage and lipid peroxidation, which promoted the generation of ferroptosis (Zhang M. et al., 2024b; Rao et al., 2023; Bai et al., 2024). Liu Y. J. et al. (2024) embedded PDA into the Fe-MOF by physical mixing, applied the prepared material to load erastin by Michael’s addition reactions, and used osteosarcoma cell membranes to wrap the surface of PDA-MOF-E, leading to the preparation of PDA-MOF-E-M. Due to the presence of the osteosarcoma cell membrane, PDA-MOF-E-M showed high biocompatibility and homologous targeting. PDA-MOF-E-M showed good stability in an acid–base environment (pH = 5.0 and pH = 9.0) and thermal environment (35°C and 42°C), indicating that it could have favorable chemical stability in various humoral environments of the human body. Fe-MOF releases a large amount of iron ions, creating an iron-rich environment in the tumor, which resulted in lipid peroxidation and promoted the generation of ferroptosis. Erastin inhibited the expression of the SLC7A11 protein, resulting in the decrease of the intracellular cysteine level and inhibition of GSH synthesis, which indirectly inhibited the activity of GPX4, led to the accumulation of lipid peroxides, and finally induced ferroptosis. Fe-MOF and erastin cooperate to promote the occurrence of ferroptosis through different mechanisms. PDA-MOF-E-M showed excellent T_1_-weighted MRI performance and enabled the combination of PTT and ferroptosis, which inhibited osteoclast differentiation and significantly inhibited the growth of osteosarcoma, providing a novel therapeutic option for the treatment of osteosarcoma (Figure 11) (Liu Y. J. et al., 2024). This study suggests that Fe-MOF@PDA was a potential ferroptosis inducer and provided a large amount of Fe^3+^, which cooperated with ferroptosis inducers that inhibit GPX4 activity to enhance ferroptosis, leading to a highly efficient anti-tumor effect.

### 2.3 Polydopamine-coated metal–organic frameworks in multimodal combination therapy

#### 2.3.1 PTT + CDT + ST

**FIGURE 12:**
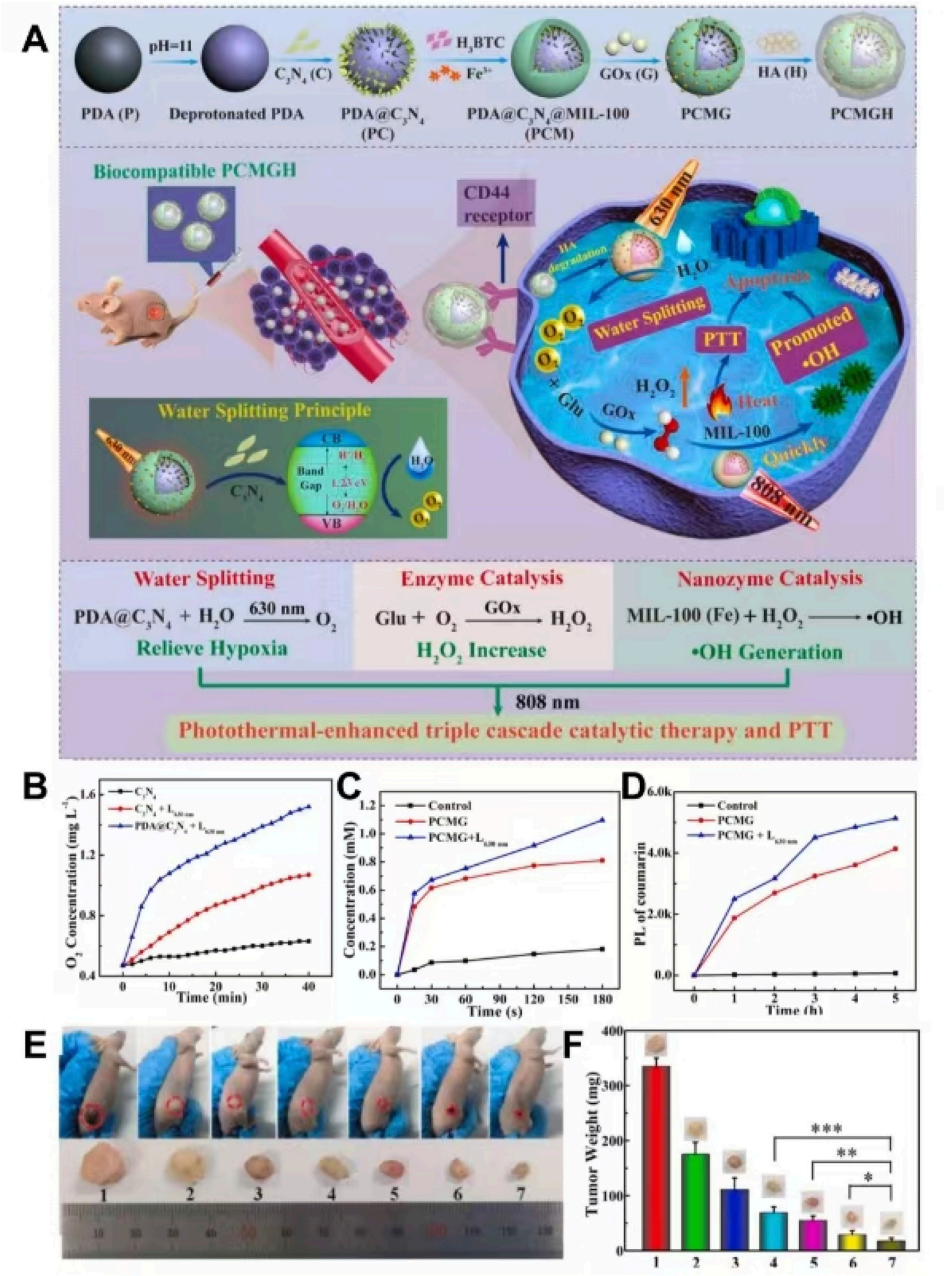
**(A)** Schematic illustration of the synthesis and anti-cancer mechanisms of PDA@C_3_N_4_@MIL@GOx@HA (PCMGH). **(B)** O_2_ generation performance in different groups. **(C)** H_2_O_2_ generation performance in different groups. **(D)** OH generation performance in different groups. **(E)** Tumor images in different treatment groups. **(F)** Tumor weight in different treatment groups. Copyright 2022, with permission from Elsevier and Yu H. et al. (2022).

GOx catalyzed glucose to produce H_2_O_2_ in the presence of O_2_, which blocked the energy supply and produced starvation therapy (ST) (Fu et al., 2023). However, the characteristics of hypoxia in the tumor microenvironment strongly restrict the therapeutic efficiency of GOx (Fu et al., 2023). The efficiency of CDT is limited due to the insufficient concentration of endogenous H_2_O_2_, which makes it impossible to use the Fenton reaction to produce a large amount of OH (Hao et al., 2023). Yu H. et al. (2022). coated carbon nitride (C_3_N_4_) on PDA by physical stirring, modified PDA@C_3_N_4_ surface with polyacrylic acid (PAA), used MIL-100 to grow on the PAA-modified PDA@C_3_N_4_ surface through layer-by-layer self-assembly, loaded GOx with shell MIL-100, and connected HA to the surface of GOx by amide bond, leading to the preparation of PDA@C_3_N_4_@MIL/GOx@HA. C_3_N_4_ cleaved water to produce O_2_, which could improve the hypoxia state of the tumor microenvironment and improve the efficiency of glucose catalyzed by GOx. GOx catalyzed glucose to produce a large amount of H_2_O_2_ in the presence of O_2_ to overcome the problem of insufficient endogenous H_2_O_2_. The iron ion in MIL-100 had peroxidase-like activity, which could catalyze H_2_O_2_ to produce toxic OH, resulting in efficient CDT. The abovementioned process was triple cascade catalysis that relieved hypoxia and produced efficient ST and CDT. PDA not only improved the O_2_ production efficiency of C_3_N_4_ but also produced a good photothermal effect, which promoted the triple cascade catalytic reaction. Due to the presence of HA, PDA@C_3_N_4_@MIL/GOx@HA could actively target tumor tissue and reduce side effects. PDA@C_3_N_4_@MIL/GOx@HA showed good biocompatibility, achieved photothermal enhanced triple cascade catalysis, and improved tumor hypoxia, enabling an efficient combination of PTT, ST, and CDT and achieving a significant inhibitory effect on hypoxic tumors (Figure 12) (Yu H. et al., 2022). This study utilized nano-enzymes to achieve a triple cascade reaction that improved hypoxia in the tumor site and overcame insufficient endogenous H_2_O_2_, which achieved highly efficient ST and CDT, acquiring a safe and efficient method of tumor therapy. Zhang et al. prepared MGH by coating hyaluronic acid-modified dopamine (HA-PDA) on the surface of MIL-100 loaded with GOx (Zhang Y.et al., 2019). Due to the presence of HA-PDA, MGH could actively target tumor tissues with high CD44 receptor expression, exhibit good biocompatibility and stability, and prevent the leakage of GOx in blood circulation. GOx catalyzed glucose in the presence of O_2_, which not only inhibited energy production and produced ST but also produced gluconic acid and H_2_O_2_. MIL-100 catalyzed H_2_O_2_ to produce OH and O_2_ through the Fenton-like reaction, which produced CDT and improved the hypoxia state of tumors. Gluconic acid deepened the local acidity of the tumor and promoted the efficiency of the Fenton-like reaction. Generated O_2_ enhanced the efficiency of glucose catalyzed by GOx. MGH produced a large amount of OH and O_2_ by cascade catalytic reaction, which enabled the efficient combination of PTT, ST, and CDT guided by PAI (Zhang Y. et al., 2019). In this study, a positive feedback cascade catalytic reaction was achieved through the combination of a nano-enzyme and a biological enzyme, resulting in the production of a large amount of H_2_O_2_ and O_2_; this approach overcame the inefficiency of CDT caused by the lack of endogenous H_2_O_2_ and the inefficiency of GOx-mediated ST caused by hypoxia, enabling the effective coordination of ST and CDT.

#### 2.3.2 PTT + CDT + chemotherapy

**FIGURE 13:**
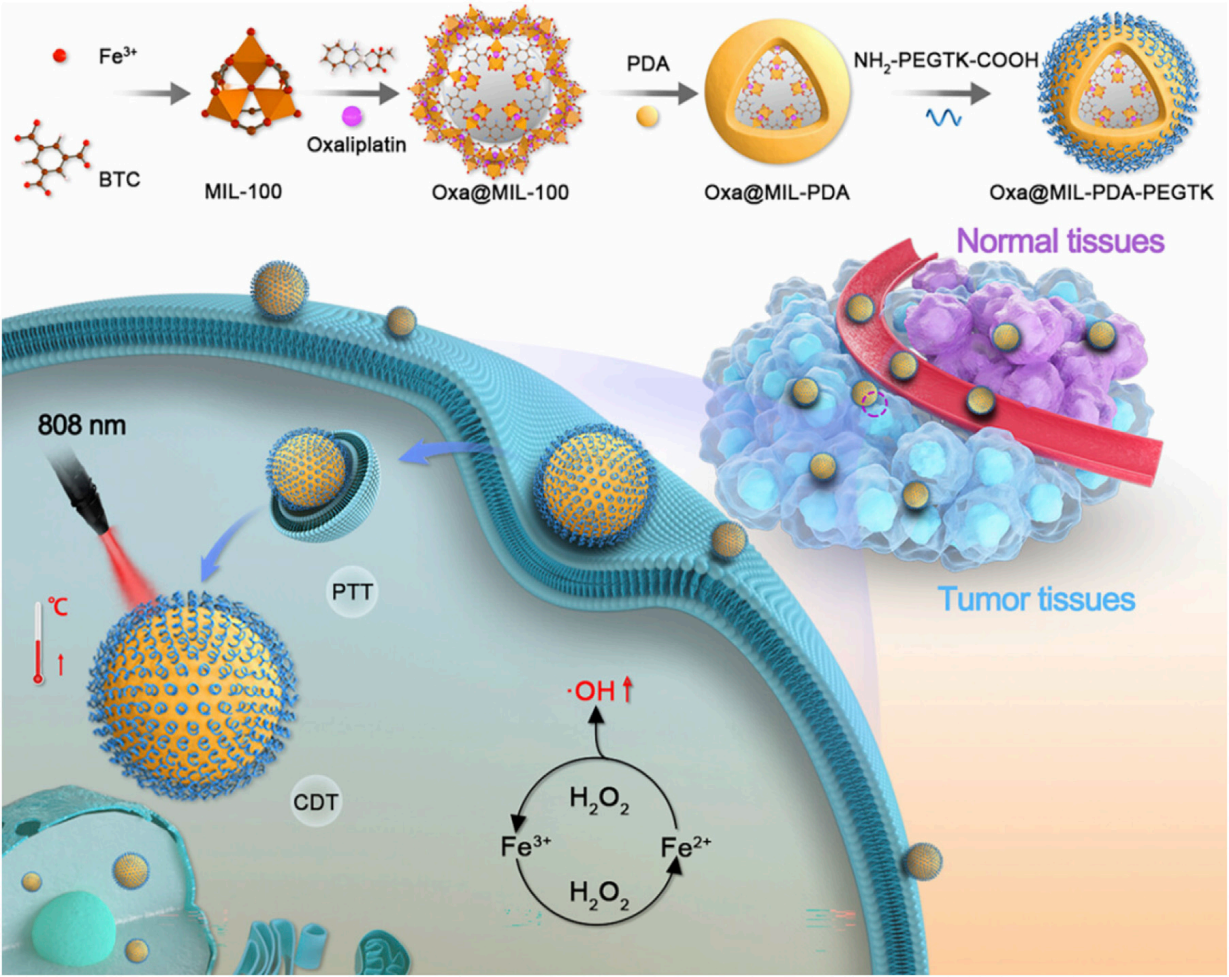
Schematic illustration of the experimental procedure for preparing Oxa@MIL-PDA-PEGTK and its applications for the efficient combination of chemodynamic therapy, chemotherapy, and photothermal therapy; copyright 2023, with permission from the American Chemical Society and Huang et al. (2023).

Huang et al. (2023) coated PDA on the surface of MIL-100 loaded with oxaliplatin (Oxa) and connected the shell PDA to NH_2_-PEGTK-COOH by amide bond to prepare Oxa@MIL-PDA-PEGTK. Oxa@MIL-PDA-PEGTK had the characteristic of acid-responsive degradation and a drug-loading capacity of 5.34%, and it enabled drug release in response to the double irritation of pH and H_2_O_2_, which was conducive to the precise release of drugs in the tumor microenvironment. Fe^2+^ in MIL-100 could react with the high concentration of H_2_O_2_ in cells to produce a large number of OH for destroying tumors, which led to CDT. Oxa@MIL-PDA-PEGTK could efficiently deliver drugs and accurately release drugs at the tumor site, enabling the combination of CDT, chemotherapy, and PTT, which significantly inhibited the growth of liver cancer (Figure 13) (Huang et al., 2023). Wang et al. synthesized Cu^2+^/ZIF-8 using a simple ion-doping method, loaded DOX with Cu^2+^/ZIF-8, and coated shell PDA on the surface of Cu^2+^/ZIF-8, leading to the preparation of DOX@Cu^2+^/ZIF-8@PDA (Wang L.et al., 2022b). DOX@Cu^2+^/ZIF-8@PDA possessed a drug-loading capacity of 9%, and it exhibited pH- and light-stimulated drug release, which reduced the side effects of chemotherapy. Released Cu^2+^ could oxidize GSH, break the redox homeostasis of tumors, and produce Cu+ to overcome the low efficiency of CDT caused by high GSH expression in the tumor microenvironment. Cu^+^ catalyzed the formation of OH from H_2_O_2_ in tumors to produce effective CDT. Due to the presence of PDA, DOX@Cu^2+^/ZIF-8@PDA had a photothermal conversion efficiency of 34.6% and showed a good photothermal effect, which enhanced the effect of CDT and chemotherapy. DOX@Cu^2+^/ZIF-8@PDA depleted GSH and achieved an efficient combination of PTT, chemotherapy, and CDT (Wang L. et al., 2022). Ren et al. (2024) coated PDA on Cu/ZIF-8 with hydroxycamptothecin (HCPT) and used erythrocyte membrane to coat on the surface of PDA, leading to the preparation of RBCM-HCPT@Cu/ZIF-8@PDA. Due to the presence of the erythrocyte membrane, RBCM-HCPT@Cu/ZIF-8@PDA could achieve immune escape and prolong blood circulation in the body, which could be efficiently enriched at the tumor site. Due to the acid-responsive degradation of ZIF-8, RBCM-HCPT@Cu/ZIF-8@PDA effectively releases copper ions and HCPT in the tumor microenvironment, thus enabling safe and effective CDT and chemotherapy. RBCM-HCPT@Cu/ZIF-8@PDA with a drug-loading capacity of 19.1% showed excellent biosafety, had acid- and light-responsive drug release, and enabled an efficient combination of CDT, chemotherapy, and PTT (Ren et al., 2024).

#### 2.3.3 PTT + PDT + CDT

**FIGURE 14:**
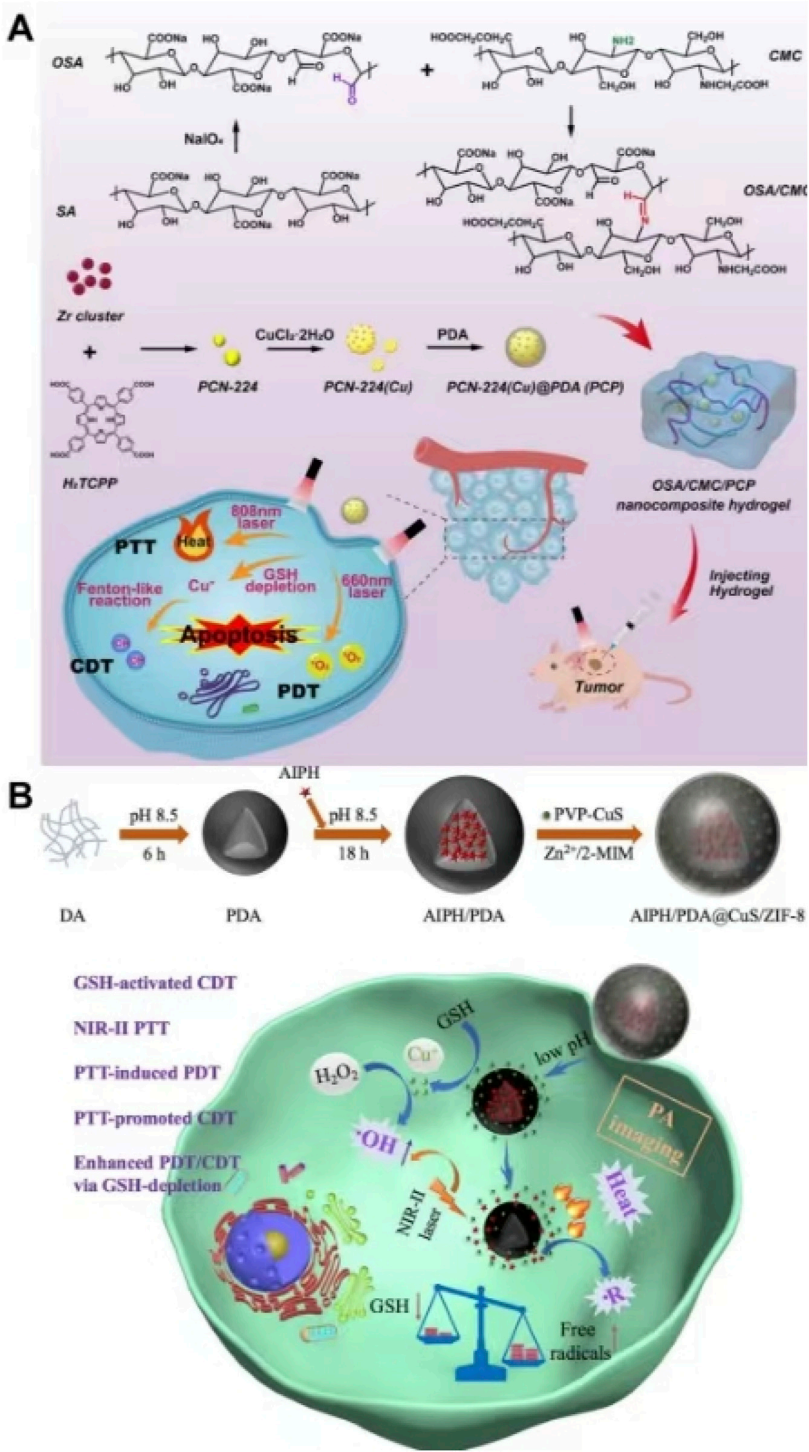
**(A)** Schematic illustration of the synthesis of OSA/CMC@PCP and its application for the synergistic therapy of breast cancer with chemodynamic therapy, photodynamic therapy, and photothermal therapy; copyright 2024, with permission from Elsevier and Zhang and Yuan (2024). **(B)** Schematic illustration of the synthesis and synergistic therapy of AIPH/PDA@CuS/ZIF-8; copyright 2022, with permission from Wiley-VCH GmbH and Zhang et al. (2021).

Due to the problems such as poor targeting, side effects, and poor accuracy of traditional treatment methods such as chemotherapy, it is very valuable to develop a non-chemotherapy system to achieve accurate and effective tumor treatment (Zhang and Yuan, 2024). Drugs or nanoparticles are usually injected into the body by subcutaneous or vascular injection and enter the tumor site through systemic blood circulation (Tu et al., 2024; Dash et al., 2024; Zuo et al., 2024). The successful enrichment of nanomaterials at tumor sites poses a significant challenge to the stability of nanoparticles *in vivo* and their tumor-targeting performance (Wang Q. et al., 2024; Zhang X. et al., 2024). Meanwhile, nanoparticles are easily cleared by the immune system, and drugs tend to leak into the bloodstream during circulation (Wang Q. et al., 2024; Zhang X. et al., 2024). Therefore, it is difficult to achieve accurate treatment of tumors. In view of the above, Zhang et al. synthesized PCN-224 (Cu) by chelating Cu^2+^ with TCPP (ligand of PCN-22) and used PDA to coat on the surface of PCN-224 (Cu) to prepare PCN-224 (Cu) @ PDA (PCP). Zhang et al. dispersed PCP into disodium alginate (OSA) and carboxymethyl chitosan (CMC) and synthesized nanocomposite self-healing hydrogels by Schiff base bonding. The complex was injected into the tumor site through intra-tumoral injection to release PCP slowly, which avoided the toxicity caused by the non-specific distribution of nanomaterials caused by systemic circulation and facilitated precise treatment. Cu^2+^ in PCN-224(Cu) reacted with GSH to produce Cu^+^, which reduced ROS scavenging and improved the efficacy of CDT and PDT. Cu^+^ reacted with a high concentration of H_2_O_2_ in tumor cells to form ROS, which could be used for CDT. PCN-224 (Cu) showed excellent performance of PDT, and PDA exhibited good photothermal performance. The complex integrated a variety of non-chemotherapy treatment methods into one platform to achieve the combined effects of PTT, PDT, and CDT, which provided a reference for the establishment of efficient, accurate, and non-chemotherapy treatment of tumors (Figure 14A) (Zhang and Yuan, 2024). The self-healing hydrogel in the study not only had superior biosafety but also had injectability, which could enter the tumor tissue through intra-tumoral injection, enabling precise drug release at the tumor site and avoiding the toxicity of drugs or nanoparticles to normal tissues during systemic circulation (Zhang and Yuan, 2024). Zhang L. et al. (2021) synthesized ZIF-8 on the surface of PDA loaded with AIPH and encapsulated PVP-modified CuS inside ZIF-8 to prepare AIPH/PDA@CuS/ZIF-8. Due to the degradation of shell ZIF-8 in an acidic environment, AIPH/PDA@CuS/ZIF-8 could accurately release CuS and AIPH in the tumor site. CuS could clear GSH and produce Cu^+^, which could react with H_2_O_2_ to form ROS through the Fenton reaction, resulting in CDT. Due to the presence of CuS, AIPH/PDA@CuS/ZIF-8 possessed a photothermal conversion efficiency of 28.05% and produced a good photothermal effect. High temperatures generated by PTT led to the decomposition of AIPH to O_2_-independent alkyl free radicals for oxygen-independent PDT. Both PDA and CuS could scavenge GSH, which reduced ROS scavenging, and disrupt the oxidative stress balance in tumor cells, improving the efficacy of PDT and CDT based on ROS as a therapeutic mechanism. AIPH/PDA@CuS/ZIF-8 achieved PAI-guided combination therapy, integrating PTT, O_2_-independent PDT, and CDT, which produced efficient anti-tumor effects on both hypoxic and normoxic tumors (Figure 14B) (Zhang L. et al., 2021). This study utilized the high temperature generated by PTT to lead to the decomposition of AIPH, resulting in the production of a large number of highly toxic alkyl radicals without the involvement of O_2_ to generate highly efficient O_2_-independent PDT, which provided a new method to overcome the inefficiency of PDT due to the lack of oxygen at the tumor site.

#### 2.3.4 PTT + PDT+ chemotherapy

**FIGURE 15:**
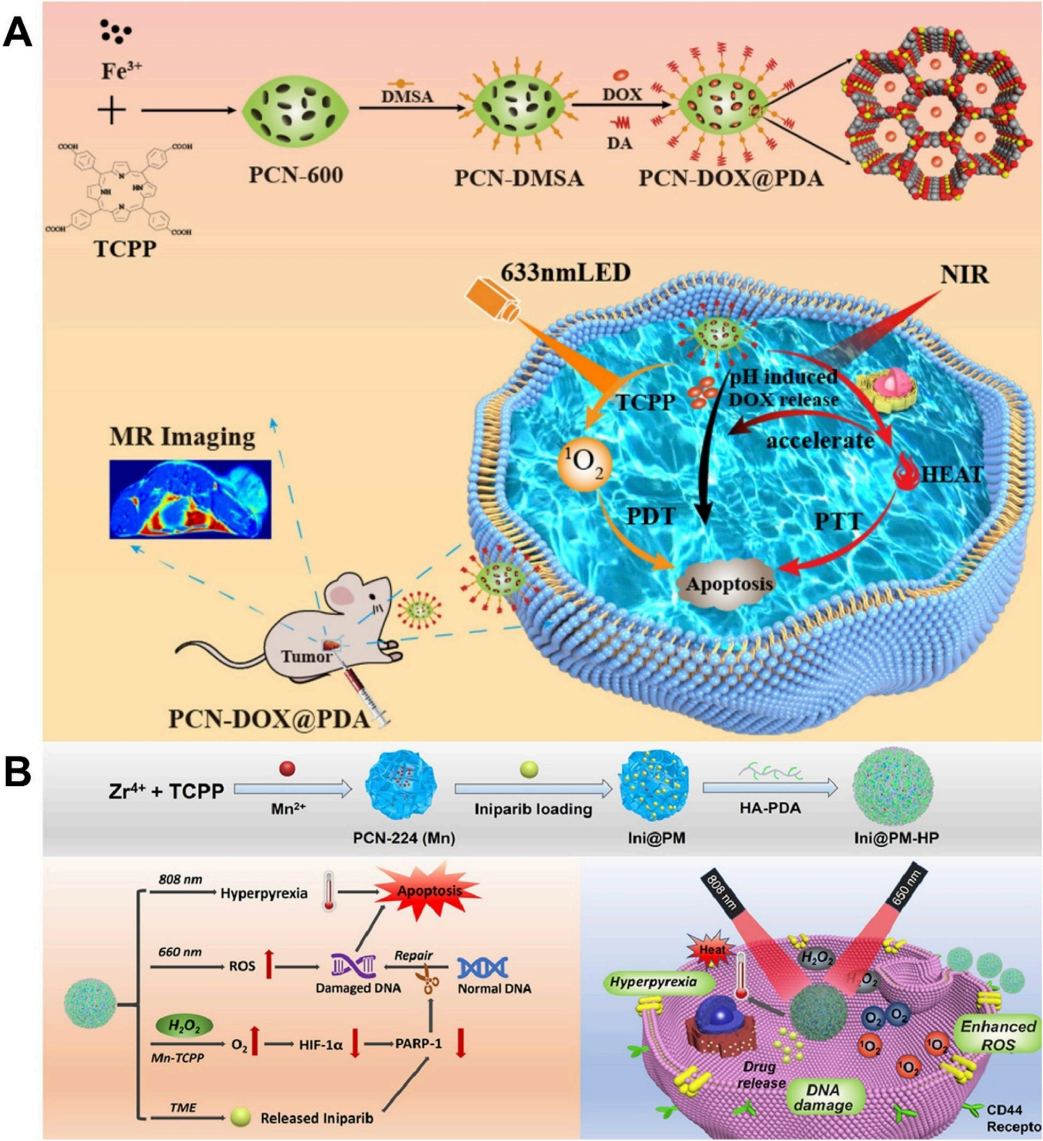
**(A)** Schematic representation of the synthesis of PCN-DOX@PDA and its application for the combined therapy of breast cancer with chemotherapy, photodynamic therapy, and photothermal therapy; copyright 2023, with permission from the American Chemical Society and Chen Z. et al. (2023). **(B)** Schematic representation of the synthesis and combined therapy of Ini@PM-HP; copyright 2022, with permission from Elsevier and Feng L. et al. (2022).

An intelligent response drug release system can improve treatment efficiency and reduce adverse reactions, which has great application potential in the accurate treatment of tumors (Zhao H. et al., 2024). Chen Z. et al. (2023) prepared PCN-DOX@PDA by loading DOX on PCN-600 assembled through the coordination of Fe^3+^ and TCPP and utilizing PDA to coat the surface of PCN-600. Due to the degradation of shell PDA in an acidic environment and the heat-promoted thermal movement of the molecules, PCN-DOX@PDA showed pH- and light-stimulation-responsive drug release, which achieved precise drug release at the tumor site and reduced the side effects of chemotherapy, promoting the implementation of precision medicine. Due to the presence of Fe^3+^ and photosensitizer TCPP, PCN-DOX@PDA exhibited superior T_2_-weighted MRI performance and efficient PDT. PCN-DOX@PDA, with a drug-loading capacity of 78%, demonstrated dual stimuli-responsive drug release and excellent biocompatibility, and it enabled an MRI-guided combination of PDT, PTT, and chemotherapy, which provided a reference for the construction of a multi-functional intelligent response drug delivery system (Figure 15A) (Chen Z. et al., 2023). Feng L. et al. (2022) prepared PCN-224 (Mn) by chelating TCPP with Mn^2+^, applied PCN-224 (Mn) to load the hydrophobic chemotherapeutic drug iniparib through electrostatic interaction, and applied HA-PDA to modify the surface of PCN-224 (Mn), leading to the preparation of Ini@PM-HP. Ini@PM-HP had a drug-loading capacity of 29.38% and could be degraded in a phosphate environment, showing phosphate-responsive drug release, which was expected to be accurately released in the tumor microenvironment with a high phosphate concentration. The released iniparib causes DNA damage and repair dysfunction, and it promoted tumor apoptosis, producing chemotherapy and improving the efficacy of PDT. Released Mn^2+^ had peroxidase-like activity, which could catalyze the high concentration of H_2_O_2_ in the tumor to produce O_2_, improving the anoxic state of the tumor and enhancing the efficacy of PDT and chemotherapy. Due to the presence of HA-PDA, Ini@PM-HP could actively target tumor tissues with high CD44 receptor expression and possessed a photothermal conversion efficiency of 19.5%, showing good photothermal performance. Ini@PM-HP used nano-enzyme to generate O_2_ *in situ*, which improved the hypoxia state of tumors and enabled a highly efficient combination of chemotherapy, PDT, and PTT, providing a multi-functional nano-platform and a reference for improving hypoxia and combined therapy (Figure 15B) (Feng L. et al., 2022).

#### 2.3.5 PTT + ST+ gas therapy

**FIGURE 16:**
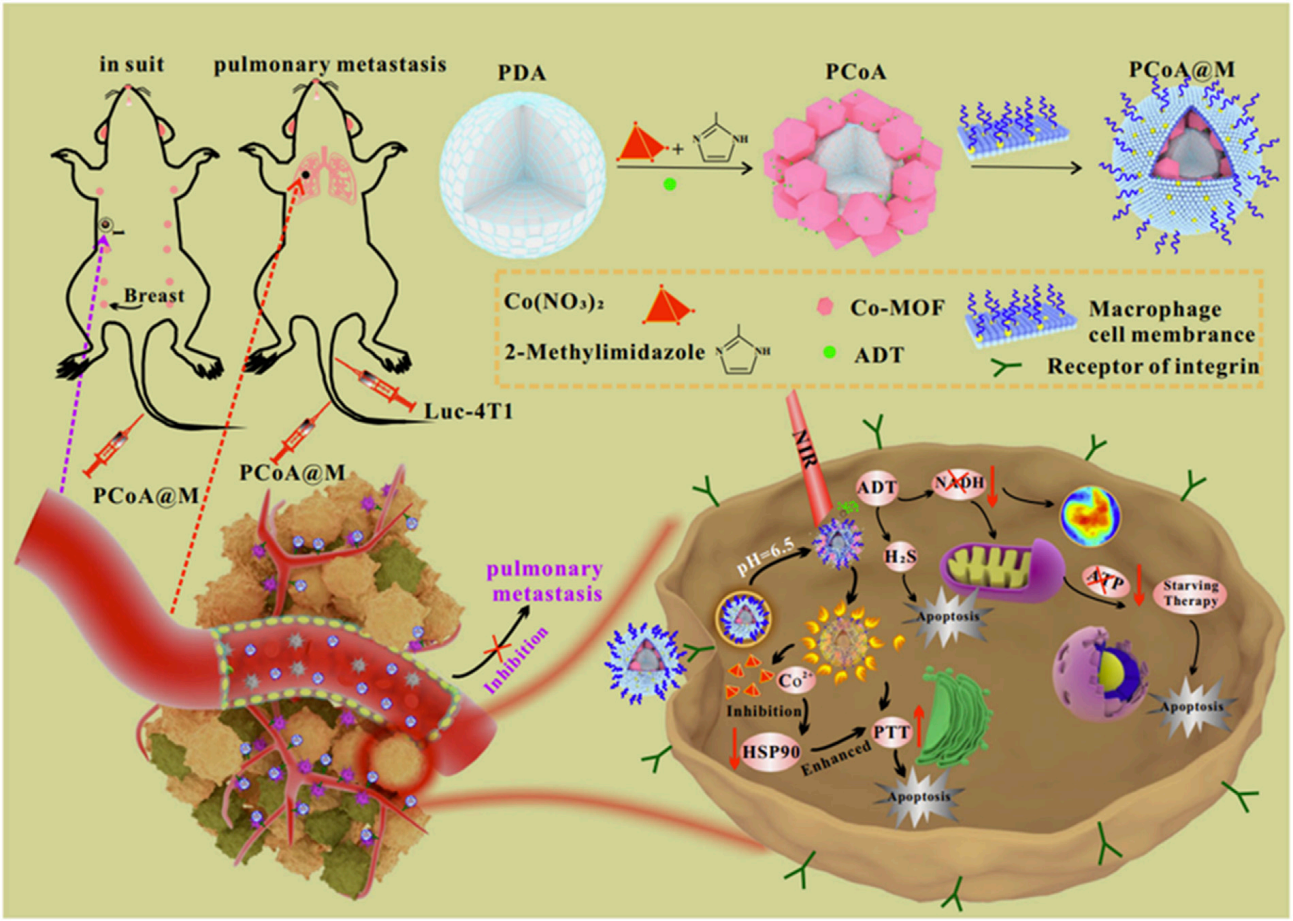
Schematic illustration of the experimental procedure for preparing PCoA@M and synergistic treatment of breast cancer induced by PCoA@M; copyright 2022, with permission from Springer Nature and Cheng K. et al. (2022). PCoA@M could be efficiently enriched at tumor sites by integrin on the membrane of macrophages. PCoA@M inhibited HSPs to enhance photothermal therapy and inhibited NADH generation, which achieved the combination of starvation therapy, gas therapy, and photothermal therapy, inhibiting tumor growth and metastasis.

H_2_S-based gas therapy is an effective anti-tumor modality, which can hinder the cell cycle, induce apoptosis of tumor cells, enhance immunotherapy, and inhibit tumor metastasis, showing a promising application (Ghaffari-Bohlouli et al., 2024; Wu G. L. et al., 2023; Li S. et al., 2023; Cheng J. et al., 2023). H_2_S has a short blood half-life and poor stability and could not specifically target tumor tissue, which severely limits the effectiveness of H_2_S treatment (Rong et al., 2022; Ge et al., 2022; Cheng K. et al., 2022). Therefore, it is of great significance to construct a multi-functional nano-platform to achieve the targeted enrichment of H_2_S at the tumor site and the accurate release of H_2_S at the tumor site (Cheng K. et al., 2022). PTT could upregulate the expression of the heat shock protein, which caused heat tolerance of tumor cells and limited the efficiency of PTT (Premji et al., 2024). Therefore, the inhibition of heat shock proteins produced during PTT is an important method to improve the efficiency of PTT (Chang et al., 2022). Due to the strong chelation of the catechol group of PDA with metal ions, Cheng K. et al. (2022) synthesized Co-MOF on the surface of PDA using the one-pot method, loaded triethole (ADT) into mesoporous Co-MOF, and coated it with a macrophage membrane, leading to the preparation of PCoA@M. Due to the expression of integrin on the membrane of macrophages, PCoA@M could recognize tumorous cells with high expression of vascular cell adhesion molecules, efficiently enrich tumor tissues, show high stability, and reduce phagocytosis of the immune system. PCoA@M was degraded in an acidic environment to achieve pH-responsive release of Co^2+^ and ADT, which facilitated accurate drug release in the acidic microenvironment of the tumor. Co^2+^ downregulated the expression of HSP90 and inhibited heat shock protein-mediated thermo-resistance in tumor cells, which increased the sensitivity of PTT. The precise release of ADT at the tumor site was catalyzed by enzymes highly expressed in the tumor cells to generate high concentrations of H_2_S, producing gas therapy. ADT reacted with nicotinamide adenine dinucleotide (NADH), leading to a reduction in the content of NADH and resulting in a dynamic imbalance in the nicotinamide adenine dinucleotide/lutein adenine dinucleotide (NADH/FAD) ratio; this disruption ultimately reduced ATP production, thereby inducing ST. NADH/FAD-mediated autofluorescence showed that the content of PCoA@M reached the maximum at 8 h after tail vein injection. PCoA@M, with a drug-loading capacity of 3.4%, showed good biosafety and enabled the combination of PTT, gas therapy, and ST, which significantly inhibited the growth and lung metastasis of breast cancer (Figure 16) (Cheng K. et al., 2022). This study utilized the Co^2+^ in PCoA@M to inhibit the expression of heat shock proteins and improve the efficiency of PTT, which suggested that Co^2+^-containing MOFs@PDA may act as a heat shock protein inhibitor, showing promising applications. This study achieved efficient enrichment of H_2_S-producing drugs at the tumor site and precise release of H_2_S-producing drugs at the tumor site, and it applied the enzymes in tumor cells to catalyze drugs to generate H_2_S, which facilitated the efficient enrichment and precise release of H_2_S at the tumor site and improved the effectiveness of H_2_S-based gas therapy, providing ideas for the efficient use of H_2_S in the treatment of tumors.

#### 2.3.6 Others

**FIGURE 17:**
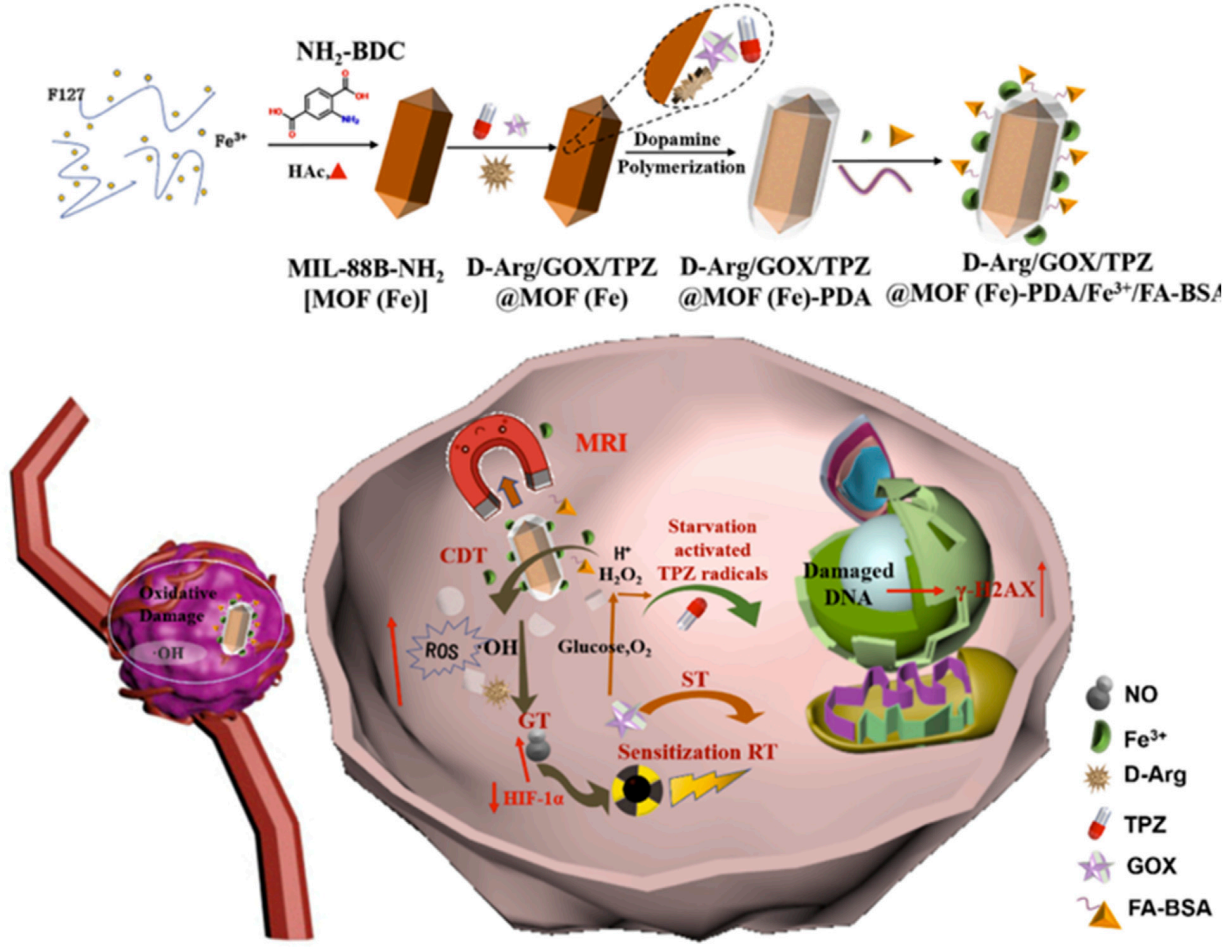
Schematic illustration of the experimental procedure for preparing D-Arg/GOX/TPZ@MOF(Fe)-PDA/Fe^3+^/FA-BSA and its application for the combined therapy of osteosarcoma with chemotherapy, starvation therapy, gas therapy, chemodynamic therapy, and radiotherapy; copyright 2023, with permission from Elsevier and Wang Y. et al. (2023b).

Zhan et al. (2023) encapsulated GOx and DOX in the inner part of ZIF-8 using the one-pot method and coated ZIF-8 with PDA to prepare DGZPNs . Because PDA and ZIF-8 were stable in a neutral environment and degraded in an acidic environment, DGZPNs not only prevented the premature release of GOx and DOX in blood circulation but also achieved acid-responsive release of GOx and DOX in the tumor microenvironment, which reduced the toxicity of drugs to normal tissues. GOx consumed glucose to disrupt the metabolic pathway of cancer cells, which led to ST. DGZPNs possessed a drug-loading capacity of 14.6% and a photothermal conversion efficiency of 36.9%, enabling the combination of chemotherapy, ST, and PTT, which could inhibit tumor growth in a safe and efficient way (Zhan et al., 2023). An G. et al. (2024) prepared MIL-101 by the solvothermal method, applied Cu nanoparticles to grow on MIL-101 through the reduction reaction, and applied Cu@MIL-101 to load cisplatin (Pt) and 1-methyl-D-tryptophan (1-MT) and connect TCPP by the amide bond, leading to the preparation of Cu@MIL-101@PMT. An et al. prepared Cu@MIL-101@PMTPC by coating PDA on the surface of Cu@MIL-101@PMT and coating CaO_2_ on the surface of PDA through physical stirring. Because PDA was coated on the surface of Cu@MIL-101 loaded with drugs, the composite avoided drug leakage in the process of blood circulation and enriched the tumor site through EPR, reducing the toxicity of drugs to normal tissues. Cu@MIL-101@PMTPC possessed a drug-loading capacity of 40.5% for Pt and 9.5% for 1-MT, and it showed pH-responsive drug release. Fe^3+^ in MIL-101 (Fe) and Cu nanoparticles could react with intracellular GSH, leading to GSH depletion, and the production of Fe^2+^ and Cu^+^, improving the efficacy of CDT and PDT. Fe^2+^ and Cu^+^ could interact with H_2_O_2_ by the Fenton-like reaction to produce ROS, which produced the effect of CDT. Outer CaO_2_ reacted with water to produce O_2_ and H_2_O_2_, which enhanced the effect of PDT and provided a continuous supply of H_2_O_2_ for Fenton-like reactions, enhancing the effect of CDT. 1-MT was an indoleamine 2-dioxygenase (IDO) inhibitor, which could enhance the expression of CD8^+^ and CD4^+^ T cells and accelerate immunogenic cell death, and it can overcome the immune escape caused by cisplatin, producing immunotherapy. Cu@MIL-101@PMTPC had cascade catalysis to achieve self-supply of O_2_ and H_2_O_2_, improving the poor efficacy of PDT caused by hypoxia and overcoming the poor efficacy of CDT caused by insufficient H_2_O_2_ in tumors. Cu@MIL-101@PMTPC enabled high-efficiency combination of CDT, PDT immunotherapy, and chemotherapy, enriching the types of multi-functional nano-platforms and providing a new strategy for cancer treatment (An G. et al., 2024). TPZ is a biological reductant with selective hypoxia cytotoxicity, which could produce highly active free radicals in a hypoxic environment, resulting in tumor cell death (Shi Q. et al., 2024; Chen Y. et al., 2024). Wang Y. et al. (2023b) used MIL-88B-NH_2_ to load D-arginine (D-Arg), GOx, and TPZ, then coated PDA on the surface of MIL-88B-NH_2_, and used shell PDA to chelate Fe^3+^ and link with folic acid-modified bovine serum albumin (FA-BSA), leading to the preparation of D-Arg/GOx/TPZ@MOF(Fe)-PDA/Fe^3+^/FA-BSA. Due to the presence of FA-BSA, the complex could actively target osteosarcoma and prolong the blood circulation time. GOx catalyzed endogenous glucose to produce H_2_O_2_ and gluconic acid, which cuts off cell energy supply, producing ST. As gluconic acid led to acidification of the tumor site, MIL-88B-NH_2_ degraded and released TPZ and Fe^3+^. Fe^3+^ reacted with H_2_O_2_ to produce ROS, which catalyzed D-Arg to produce NO, enhancing the efficacy of radiotherapy (RT) and generating gas therapy. Endogenous glucose catalyzed by GOx consumed O_2_ and RT consumed O_2_, resulting in serious hypoxia of the tumor microenvironment. The serious hypoxia in the tumor site could activate TPZ, resulting in efficient chemotherapy. The shell PDA could not only seal the MIL-88B-NH_2_ pores loaded with drugs but also chelate Fe^3+^ to produce superior T_1_-weighted MRI performance. The complex showed pH- responsive drug release and enabled the combination of ST, gas therapy, CDT, low-dose RT, and chemotherapy, which significantly inhibited tumor growth and lung metastasis (Figure 17) (Wang Y. et al., 2023b). This study utilized ST and RT to lead to significant hypoxia at the tumor site, which activated hypoxia-activated drugs to produce highly effective anti-tumor effects, providing a new strategy for treating tumors by making use of the characteristics of hypoxia at the tumor site and offering an effective method to overcome treatment resistance due to hypoxia.

## 3 Summary and challenges

MOFs@PDA are classical multifunctional nanomaterials and ideal carriers for constructing nano-drug delivery systems, achieving integrated diagnosis and treatment and comprehensive treatment. MOFs@PDA have made breakthrough progress in solving problems such as treatment resistance caused by hypoxia at the tumor site, low efficiency of low-temperature PTT caused by the upregulation of heat shock proteins, chemotherapy resistance, and low efficiency of CDT caused by high GSH expression inside the tumor. MOFs@PDA could internally encapsulate drugs and efficiently load drugs on the surface using various mechanisms and could achieve pH- and light-stimulation-responsive drug release, which facilitated the precise release of drugs at the tumor site, improved the efficiency of chemotherapy, and reduced the side effects of chemotherapy. Due to the excellent photothermal properties of PDA and the good imaging properties of MOFs, MOFs@PDA are not only excellent multifunctional photothermal agents that combine diagnostic and photothermal properties but can also be loaded with drugs or enzymes, which makes it easy to achieve comprehensive treatment. MOFs@PDA not only scavenge GSH to break redox homeostasis but also catalyze the generation of ROS from H_2_O_2_, which sensitize the effects of various tumor-treatment modalities. Therefore, MOFs@PDA are of great value for biomedical applications.

MOFs@PDA also face great challenges in clinical translation. First, the safety of MOFs@PDA is the basis for its clinical translational application. However, the current safety assessment of MOFs@PDA is mostly limited to short-term toxicity, such as the effect on body weight and pathological changes in major organs in animals. The lack of assessment of long-term toxicity of MOFs@PDA limits the clinical translational application of MOFs@PDA. When selecting metal ions and organic ligands to compose MOFs, we can pick the metal ions essential for the human body and FDA-approved organic ligands, thus guaranteeing the safety of MOFs@PDA from the beginning of the experiment. Second, the preparation process of MOFs@PDA is relatively complex, which is not conducive to the reproducibility of samples and could not provide the availability of a large number of samples for biomedical applications. The optimization of experimental parameters and the standardization of experimental steps for the preparation of simple and multifunctional MOFs@PDA are of great value for the diagnosis and treatment of tumors. Third, some MOFs@PDA with PDA as nuclei and MOFs as shells may be unstable in the physiological environment, which can be easily aggregated and phagocytosed after the conditioning effect of the immune system and are unable to be effectively enriched at the tumor site. Researchers can utilize active targeting molecules, cell membranes, and albumin for the surface modification of MOFs@PDA to improve the water solubility and targeting of MOFs@PDA. Fourth, the lack of in-depth and systematic studies on the degradation mechanism and pharmacokinetic characteristics of MOFs@PDA *in vivo* is not conducive to selecting the optimal therapeutic dosage of MOFs@PDA and understanding the *in vivo* clearance of MOFs@PDA, which makes it impossible to rationally utilize MOFs@PDA to treat tumors. Researchers need to monitor the metabolism of MOFs@PDA in large primates for a long time and carefully study the specific degradation mechanism of MOFs@PDA. Fifth, the specific molecular mechanisms and signaling pathways of MOFs@PDA’s anti-tumor effects are not clear, which is not conducive to guiding clinical medication. Researchers can explore the specific molecular mechanism of MOFs@PDA’s anti-tumor effects by sequencing the genome of MOFs@PDA-treated cells and employing a large number of biological experiments. For the clinical translation of MOFs@PDA, researchers need to take a rational approach to address the safety, stability, and targeting and scale-up preparation of MOFs@PDA and deeply investigate the metabolic mechanism and specific anti-tumor mechanism of MOFs@PDA.

In summary, this paper summarized the progress made by MOFs@PDA in the field of tumor diagnosis and treatment in a timely manner, with the expectation of promoting the biomedical application of MOFs@PDA, increasing the development of novel strategies for tumor diagnosis and treatment, and inspiring researchers to explore the clinical translational applications of MOFs@PDA. Despite the significant challenges, MOFs@PDA will continue to make groundbreaking research advances in tumor diagnosis and treatment and promote the clinical translational application of nanomedicine.
